# Gpr35 Expression Mitigates Neuroinflammation and Enriches Gut *Lactobacillus* to Relieve Parkinson’s Disease

**DOI:** 10.34133/research.0846

**Published:** 2025-08-25

**Authors:** Tianyu Meng, Yufei Zhang, Shoupeng Fu, Shaohua Ma

**Affiliations:** ^1^ Institute of Biopharmaceutical and Health Engineering, Shenzhen International Graduate School (SIGS), Tsinghua University, Shenzhen 518055, China.; ^2^ Key Laboratory of Industrial Biocatalysis, Ministry of Education, Tsinghua University, Beijing 100084, China.; ^3^ Key Lab of Active Proteins and Peptides Green Biomanufacturing of Guangdong Higher Education Institutes, Tsinghua Shenzhen International Graduate School, Shenzhen 518055, China.; ^4^ College of Veterinary Medicine, Jilin University, Changchun 130062, China.

## Abstract

Parkinson’s disease (PD) is associated with gut–brain axis and gut microbiota alterations, but the functioning mechanism remains to be elucidated. In this study, we identified G protein-coupled receptor 35 (Gpr35) as a key regulator for the gut–brain association under the PD context. It investigated the impact of Gpr35 deficiency on motor function, neuroinflammation, and dopaminergic neurodegeneration, using the Gpr35 knockout (Gpr35^−/−^) and wild-type (WT) mice in a 1-methyl-4-phenyl-1,2,3,6-tetrahydropyridine (MPTP)-induced PD model, and Gpr35 up-/down-regulation on reverse neuroinflammation, oxidative stress, and neuronal apoptosis using Gpr35 agonist kynurenic acid (KYNA) and small interfering RNA in microglial and dopaminergic cell models. It was confirmed that Gpr35 may prevent PD by modulating neuroinflammation and gut microbiota and metabolite composition, specifically through enriching *Lactobacillus*, and substantially regulating tyrosine metabolism, neuroactive ligand–receptor interaction, and tryptophan metabolism pathways, thereby inhibiting the progression of PD. Our findings highlight the potential of targeting Gpr35 to influence both the gut microbiota and central nervous system, offering new insights into the treatment of PD.

## Introduction

Parkinson’s disease (PD) is a progressive neurodegenerative disorder predominantly affecting aging populations, characterized by the loss of dopaminergic neurons and the pathological accumulation of Lewy bodies in the substantia nigra (SN) pars compacta region [1]. While the precise etiology of PD remains multifaceted, around 10% of cases are associated with genetic factors like α-synuclein (α-syn) gene alterations, whereas the majority (90%) are idiopathic, with multifactorial causes involving genetic susceptibility, environmental exposures, and aging [2]. Recent studies have identified several pathological mechanisms contributing to PD pathogenesis, including impaired mitochondrial function, elevated oxidative stress levels, abnormal protein accumulation, and chronic inflammatory responses within neural tissue [3–6]. Emerging research highlights neuroinflammation as a central mechanism driving PD advancement, with immune system dysregulation intensifying the interplay between genetic predispositions and environmental triggers [7].

Recent investigations into the gut–brain axis have highlighted the critical involvement of intestinal microorganisms in PD development [8]. PD patients frequently exhibit gut microbiota dysbiosis, marked by reduced beneficial bacteria (e.g., *Faecalibacterium*) and increased potentially pathogenic species [9–11]. Such dysregulation results in diminished synthesis of short-chain fatty acids (SCFAs), which subsequently compromises intestinal barrier function and facilitates the translocation of pro-inflammatory factors into the central nervous system (CNS) [12]. Dysregulated gut microbiota and metabolites may disrupt intestinal epithelial barrier function, increase permeability, and trigger neuroinflammation, thereby contributing to dopaminergic neuron degeneration [8]. Microglia, the key immune cells of the CNS, are important mediators of neuroinflammation and synaptic regulation [13]. Contemporary findings reveal that intestinal flora substantially modulates microglial behavior and CNS immune responses, highlighting promising intervention strategies through microbial manipulation for neurological disorders [14].

G protein-coupled receptors (GPCRs) are widely recognized as the most prominent group of therapeutic targets, playing critical functions in both disease mechanisms and treatment strategies [15,16]. As a class A rhodopsin-like orphan GPCR, G protein-coupled receptor 35 (Gpr35) has been implicated in multiple disease pathways, including gastrointestinal dysfunction, cardiovascular pathology, and oncogenesis [17–19]. Gpr35 exhibits high expression levels in gastrointestinal epithelial cells (stomach, small intestine, and colon) as well as immune cells including dendritic cells and macrophages. Through these cellular distributions, it plays a crucial role in preserving intestinal equilibrium while integrating metabolic, immunological, and microbiological signals [20]. Notably, Gpr35 expression is increased under inflammatory and hypoxic conditions, such as myocardial infarction and inflammatory bowel disease [21,22]. As an endogenous ligand of Gpr35, kynurenic acid (KYNA) has been well documented for its role in tryptophan metabolism and its implications in neurological disorders [23]. Despite its established role in immune regulation, the involvement of Gpr35 in PD remains unexplored.

Given the critical role of Gpr35 in immune modulation and its expression in the gut epithelium, coupled with the established significance of the gut–brain axis in PD regulation [8], our study provides the first evidence suggesting that Gpr35 could modulate PD progression via microbiota–neuroimmune crosstalk. Therefore, we investigate the role of Gpr35 in PD pathogenesis, focusing on its impact on neuroinflammation, dopaminergic neurodegeneration, and gut microbiota composition. Our data suggest that Gpr35 deficiency exacerbates PD-related motor deficits, neuroinflammation, and dopaminergic neuron loss, while activation of Gpr35 by its agonist KYNA exerts neuroprotective and anti-inflammatory effects. Furthermore, we reveal that Gpr35 modulates gut microbiota composition, particularly affecting *Lactobacillus* abundance, which may contribute to PD pathogenesis. These results position Gpr35 as a promising therapeutic target for PD, warranting further exploration of its molecular mechanisms and clinical potential.

## Results

### Gpr35 knockout (Gpr35^−/−^) exacerbates the progression of PD and neuroinflammation in the SN

**Fig. 1.**
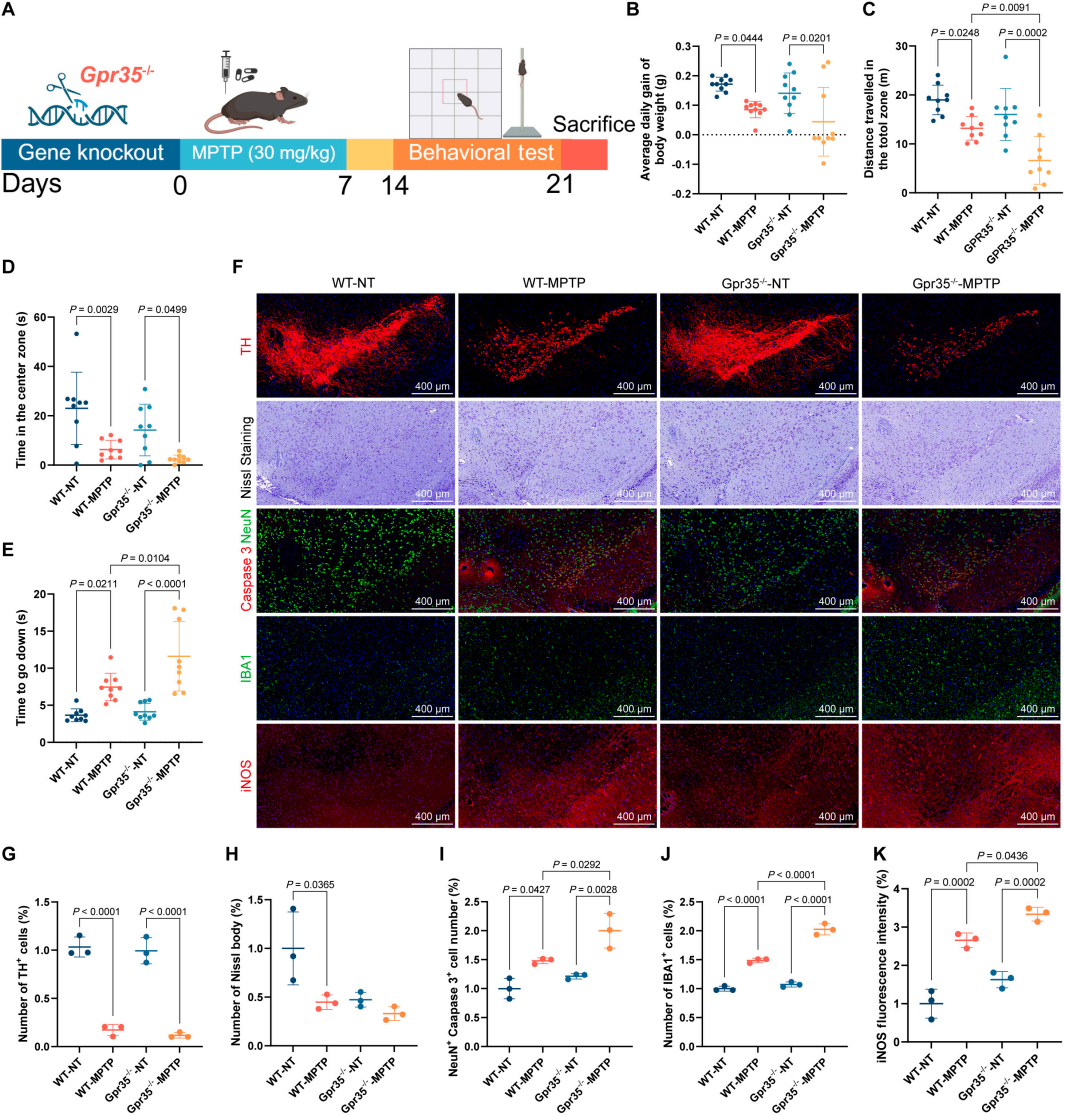
Gpr35^−/−^ exacerbates PD progression and inflammation in the SN. (A) Schematic diagram illustrating the generation of Gpr35^−/−^ mice and the establishment of the PD model. (B) The average daily weight gain during the observation period was quantified (*n* = 10 biologically independent mice). (C) The total distance traveled in the open-field test was also quantified (*n* = 9 biologically independent mice). (D) The time mice spent crossing the central zone in the open-field test was recorded (*n* = 9 biologically independent mice). (E) Time required for mice to climb down from the top during the pole test (*n* = 9 biologically independent mice). (F) Representative fluorescence staining images of TH, Nissl bodies, Caspase 3^+^ NeuN^+^ cells, microglial marker IBA1, and iNOS in the SN (from top to bottom). Scale bars, 400 μm. (G to J) Quantitative analysis of fluorescence staining results showing the number of TH^+^ cells (G), Nissl bodies (H), NeuN^+^ Caspase 3^+^ cells (I), and IBA1^+^ cells (J), compared to the WT-NT group (*n* = 3 biologically independent mice). (K) Quantification of the mean fluorescence intensity of iNOS compared to the WT-NT group (*n* = 3 biologically independent mice). Data are presented as the mean ± SEM.

Firstly, we compared the behavioral performance between the wild-type (WT), negative control (WN) group and the Gpr35^−/−^, nontreated (GN) group. Figure 1A illustrates the experimental workflow for animal studies. The analysis revealed comparable body weight fluctuations between groups (Fig. 1B), with no statistically significant variations observed, movement trajectories (Fig. S1A), or other quantitative parameters [including the time movement in the central area (Fig. 1D) and total travel distance (Fig. 1C)] in the open-field test and time it took the mouse to get to the bottom of the pole in the pole test (Fig. 1E). However, 1-methyl-4-phenyl-1,2,3,6-tetrahydropyridine (MPTP) injection led to decreased weight growth (Fig. 1B) and motor dysfunction (Fig. 1C to E) in both WT and Gpr35^−/−^ mice, with more pronounced impairments observed in the Gpr35^−/−^-MPTP (GM) group compared to the WT-MPTP (WM) group. Specifically, Gpr35^−/−^ PD mice exhibited a shorter total travel distance in the open-field test (Fig. 1C) and longer times in the pole test (Fig. 1E), indicating more severe motor deficits. Additionally, tyrosine hydroxylase (TH; key marker molecules of midbrain dopaminergic neurons) expression was significantly decreased in both WT and Gpr35^−/−^ mice following MPTP injection (Fig. 1F and G, SEM), consistent with selective dopaminergic neuron loss in the SN. However, the number of Nissl bodies (biomarkers of neuronal functional status) showed no significant changes in the Gpr35^−/−^ mice (Fig. 1F and H). Quantitative analysis revealed that MPTP administration significantly elevated midbrain neuronal apoptosis, as evidenced by increased NeuN^+^/caspase 3^+^ double-positive cells. Importantly, Gpr35^−/−^ PD mice exhibited substantially exacerbated apoptotic responses compared to WT PD controls (Fig. 1F and I). Finally, we evaluated the inflammatory state. MPTP-induced PD mice displayed excessive microglial activation [ionized calcium binding adapter protein 1 (IBA1^+^), microglial activation markers] and elevated levels of the stress inflammatory enzymes inducible nitric oxide synthase (iNOS; inflammatory biomarkers). Notably, Gpr35^−/−^ further exacerbated microglial activation and iNOS expression (Fig. 1F, J, and K). Taken together, the data establish that genetic deletion of Gpr35 worsens key PD-related phenotypes in the MPTP model, particularly affecting motor performance, survival of dopaminergic neurons, and the progression of neuroinflammation.

### Gpr35^−/−^ restructures gut microbial composition and metabolite profiles in WT and PD mouse models

**Fig. 2.**
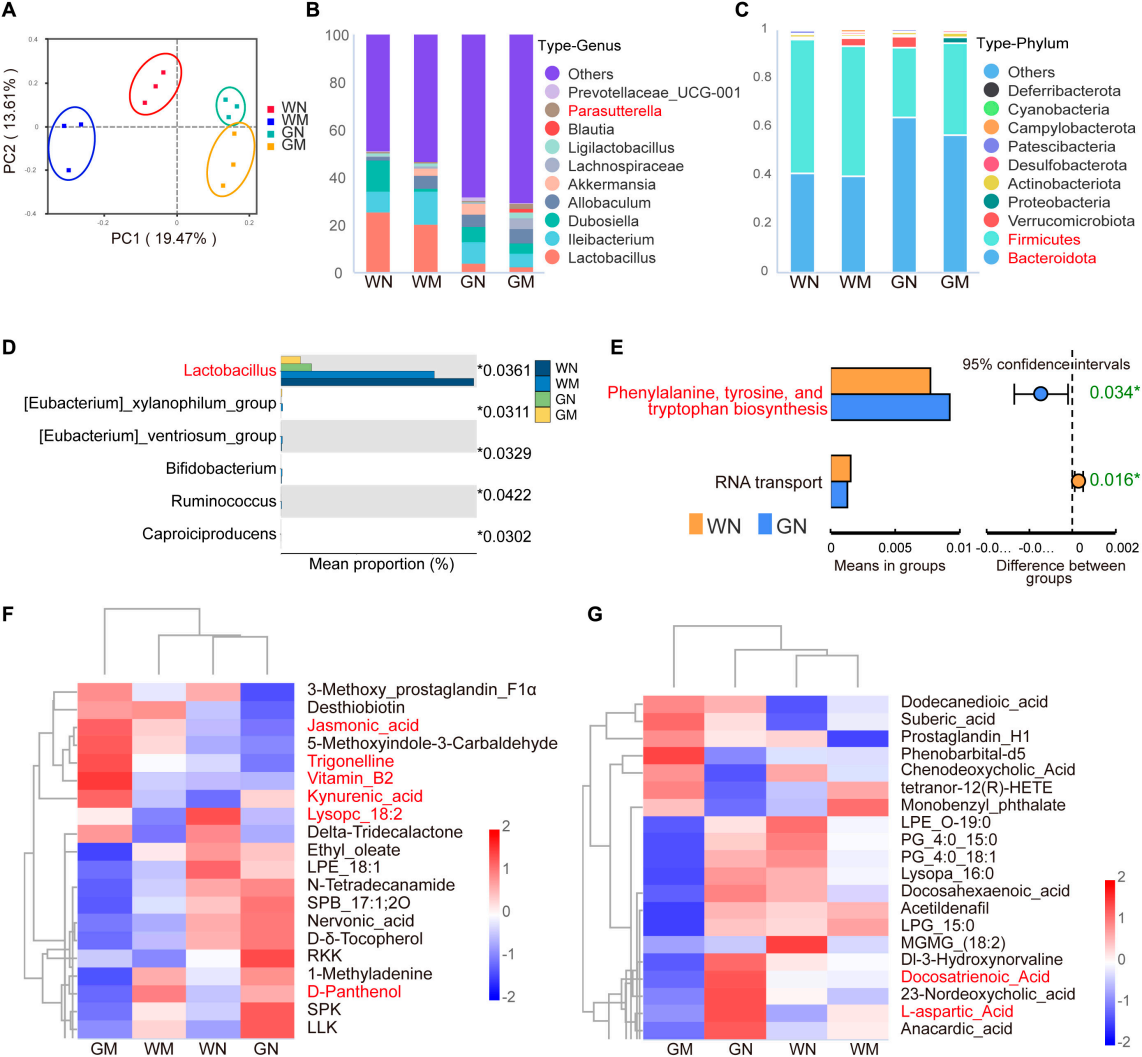
Gpr35 ablation alters gut microbiota and metabolite composition in WT and PD mouse models. (A) β-Diversity of the gut microbiome from the cecal contents of WT and Gpr35^−/−^ mice (*n* = 3 biologically independent mice) as determined by PCoA of Jaccard distances. (B and C) Average relative abundance of bacteria at the genus (B) and phylum (C) levels. (D) Screening results of differential microbial communities by Kruskal–Wallis test (^*^P < 0.05). (E) Significance analysis plots of differential functional pathways between WN versus GN groups (^*^P < 0.05 indicates a significant difference). (F and G) Heatmaps of differential metabolite clustering in positive (F) and negative (G) modes. The relative quantitative values of all differential metabolites were log_10_-transformed. Differential metabolites were screened based on the criteria of VIP > 1.0, fold change (FC) > 1.2 or FC < 0.833, and *P* < 0.05. WN, wild-type negative control mice; WM, wild-type MPTP-treated mice; GN, Gpr35^−/−^ negative control mice; GM, Gpr35^−/−^ MPTP-treated mice.

To elucidate the mechanism through which Gpr35 deletion exacerbates PD progression, we investigated its impact on gastrointestinal homeostasis, given its high expression in the gastrointestinal tract. We performed 16*S* ribosomal DNA identification analysis on 1,099 operational taxonomic units (OTUs). Microbial diversity analysis using the Shannon–Wiener index revealed comparable species-level distribution patterns among all 4 experimental groups (Fig. S2A). β-Diversity analysis revealed community composition difference through principal coordinate analysis (PCoA) and nonmetric multi-dimensional scaling (NMDS) (Fig. 2A and Fig. S2B). *Parasutterella*, a genus associated with neurological disorders including schizophrenia and cerebral palsy [24,25], was highest in the GM group (Fig. 2B). At the phylum level, *Bacteroidota* and *Firmicutes* predominated, while *Proteobacteria*, containing numerous pathogenic species, were most abundant in the GM group (Fig. 2C). The Kruskal–Wallis test and linear discriminant analysis (LDA) both indicated that the relative abundance of *Lactobacillus* was significantly lower in Gpr35^−/−^ mice than in WT controls, with an additional decline under PD conditions (Fig. 2D and Fig. 2D, E, and G), highlighting its potential therapeutic implications in PD pathogenesis. In contrast to the WN and WM groups where *Akkermansia* and *Verrucomicrobiale*s were the predominant differential bacteria, Gpr35 knockout PD mice exhibited significantly elevated levels of *Clostridia* and *Lachnospirales* compared to normal Gpr35^−/−^ mice in the GM group (Fig. S2J and K). Subsequently, we colonized *Lactobacillus* in the PD mouse model (Fig. S1E) and found that it could greatly alleviate motor dysfunction in the PD mice (Fig. S1F to K). Functional profiling of microbial communities was performed using PICRUSt2 (Phylogenetic Investigation of Communities by Reconstruction of Unobserved States, version 2), which predicts metabolic potential from 16*S* ribosomal RNA (rRNA) gene sequences. At the third functional tier, significant changes in RNA transport, phenylalanine, tyrosine, and tryptophan biosynthesis were observed in WT mice before and after Gpr35^−/−^ (Fig. 2E).

Untargeted metabolomic profiling identified lipids/lipid-like compounds and organic acids as predominant gut metabolites (Fig. S2C). KEGG (Kyoto Encyclopedia of Genes and Genomes) pathway mapping demonstrated their primary involvement in 3 metabolic processes: amino acid metabolism (111 compounds), lipid metabolism (105 compounds), and digestive system regulation (62 compounds) (Fig. S2F). Variable importance in projection (VIP) analysis identified metabolomic signatures relevant to PD pathogenesis, showing marked intergroup differences. These included l-aspartic acid (a neurotransmitter-related metabolite), docosahexaenoic acid (involved in lipid metabolism), trigonelline (associated with oxidative stress and inflammatory responses), jasmonic acid (a plant hormone with anti-inflammatory properties), d-panthenol (related to mitochondrial energy metabolism), and Lysop 18:2 (implicated in sphingolipid metabolism pathways) (Fig. 2F and G). It is noteworthy that KYNA levels were also significantly elevated following Gpr35 gene knockout. Functional annotation of differential metabolites between GN and WN groups through KEGG analysis revealed predominant associations with digestive processes, amino acid metabolic pathways, and membrane transport mechanisms (Fig. S2H). Metabolic pathway analysis identified significant enrichment in linoleic acid metabolism, specific amino acid pathways (including glutathione and arginine/proline metabolism), and adenosine triphosphate (ATP)-binding cassette (ABC) transporter systems (Fig. S2I). GSEA (gene set enrichment analysis) further demonstrated marked involvement of tyrosine/tryptophan metabolism (showing substantial enrichment in the GM group compared with WM) and neuroactive ligand–receptor interactions in both WT and Gpr35^−/−^ PD models (Fig. S3A to C). The data presented in Fig. S1 and Fig. 2D and E collectively indicate that Gpr35 deletion decreases *Lactobacillus* abundance and perturbs tryptophan metabolism, suggesting its possible involvement in PD development via this metabolic pathway.

To elucidate the complex interplay between microbial communities and metabolism, we conducted an integrated analysis (Fig. S3D). The relative abundance of *Eubacterium_ventriosum* correlates with multiple metabolites. Analysis of WN versus WM groups revealed significant associations between *Rikenellaceae* and *Family_XIII_AD3011* abundance patterns and specific metabolic profiles.

Our findings include reduced *Lactobacillus* and increased *Proteobacteria* in Gpr35^−/−^ mice, along with substantial alterations in metabolites related to neurotransmitters and lipid metabolism, along with relevant biochemical pathways including tyrosine metabolic processes and neuroactive ligand–receptor signaling cascades and tryptophan metabolism pathways. Integrated analysis reveals robust associations between particular microbial species and metabolic profiles, indicating that Gpr35 likely modulates PD progression via microbiota–metabolite crosstalk.

### FMT treatment can reduce neuroinflammation and PD-related pathological changes in SN

**Fig. 3.**
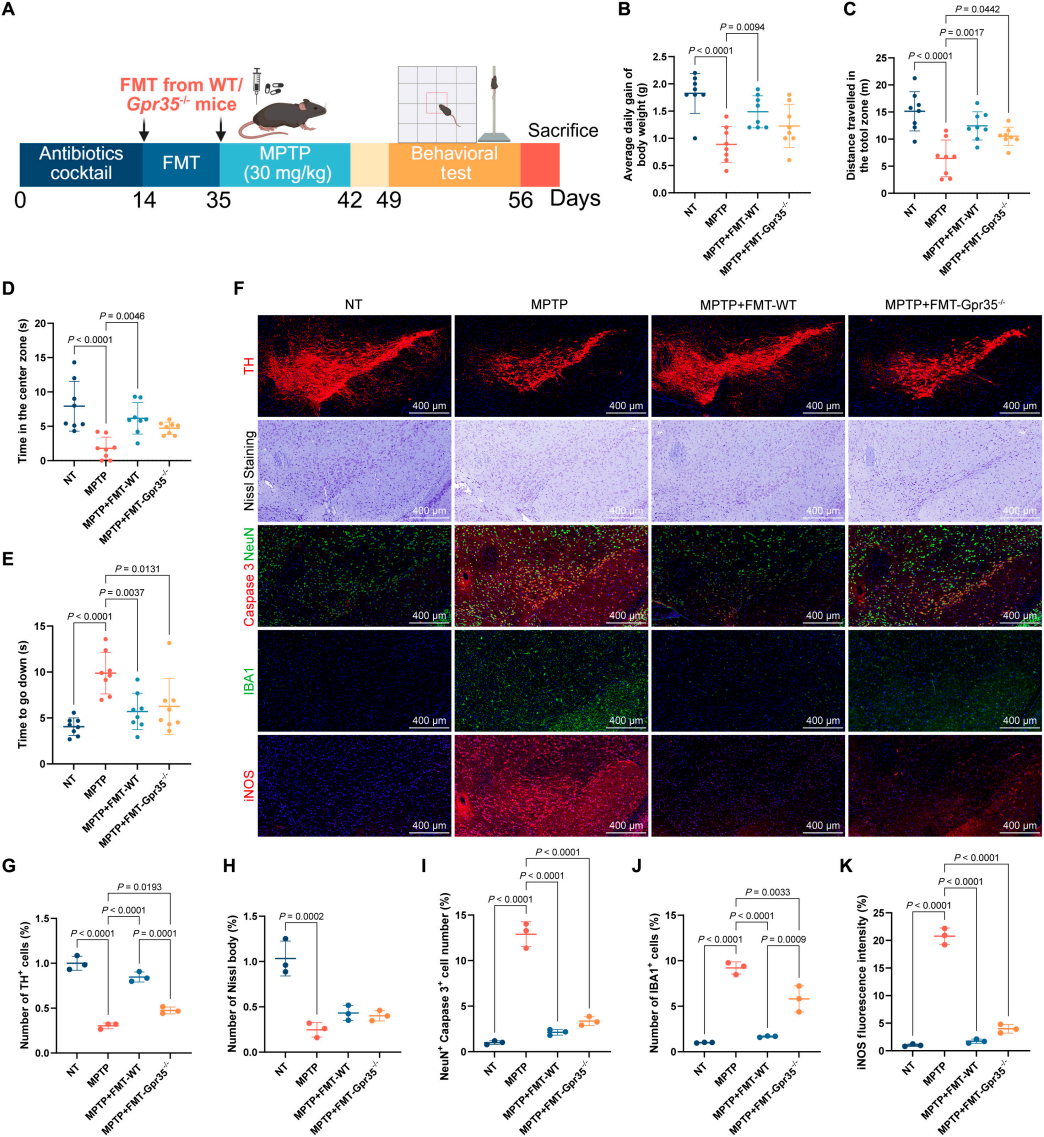
FMT administration alleviates PD-associated histological alterations and inflammation in the SN. (A) Schematic representation of the FMT procedure. (B) The average daily weight gain during this period was quantified (*n* = 8 biologically independent mice). (C and D) Quantitative analysis of open-field test results: Time spent crossing the central zone (D) and total distance traveled (C) were recorded (*n* = 8 biologically independent mice). (E) Time required for mice to climb down from the top in the pole test (*n* = 8 biologically independent mice). (F) Representative fluorescence staining images of TH, Nissl bodies, NeuN^+^ Caspase 3^+^ cells, microglial marker IBA1, and iNOS in the SN (top to bottom). Scale bars, 400 μm. (G to J) Quantitative analysis of fluorescence staining results: The number of TH^+^ cells (G), Nissl bodies (H), NeuN^+^ Caspase 3^+^ cells (I), and IBA1^+^ cells (J) was calculated (*n* = 3 biologically independent mice). (K) Quantification of mean fluorescence intensity of iNOS, normalized to the NT group (*n* = 3 biologically independent mice). Data are presented as mean ± SEM.

To further explore whether intestinal flora mediates the behavioral and phenotypic abnormalities observed in Gpr35^−/−^ mice, as well as the potential role of gut flora in alleviating PD symptoms, we conducted fecal microbiota transplantation (FMT). The effect of bacterial colony removal is shown in Fig. S4. Figure 3A outlines the experimental protocol for the FMT study. PD mice treated with negative control (NT) mouse microbiota showed significant weight recovery, while no significant weight gain was observed in PD mice treated with Gpr35^−/−^ mouse microbiota (Fig. 3B and Fig. S1D). Both FMT-treated groups showed behavioral improvements (Fig. 3C to E and Fig. S1C). Histological analyses revealed that FMT treatment elevated counts of TH-immunoreactive neurons within the SN region (Fig. 3F and G) and reduced neuronal apoptosis (Fig. 3F and I). At the same time, there was no significant change in the number of Nissl bodies (Fig. 3F and H). FMT also attenuated IBA1^+^ microglial cells (Fig. 3F and J) and reduced expression of iNOS (Fig. 3F and K). Further analysis revealed that the therapeutic effects of microbiota from NT mice were more pronounced than those from Gpr35^−/−^ mice. These findings indicate that intestinal microbiota substantially influences PD pathogenesis, with Gpr35-modulated microbial populations mediating improvements in both behavioral deficits and neuropathological features.

### Gpr35 regulates intestinal structure and barrier integrity in both MPTP-induced and FMT-treated mouse models

**Fig. 4.**
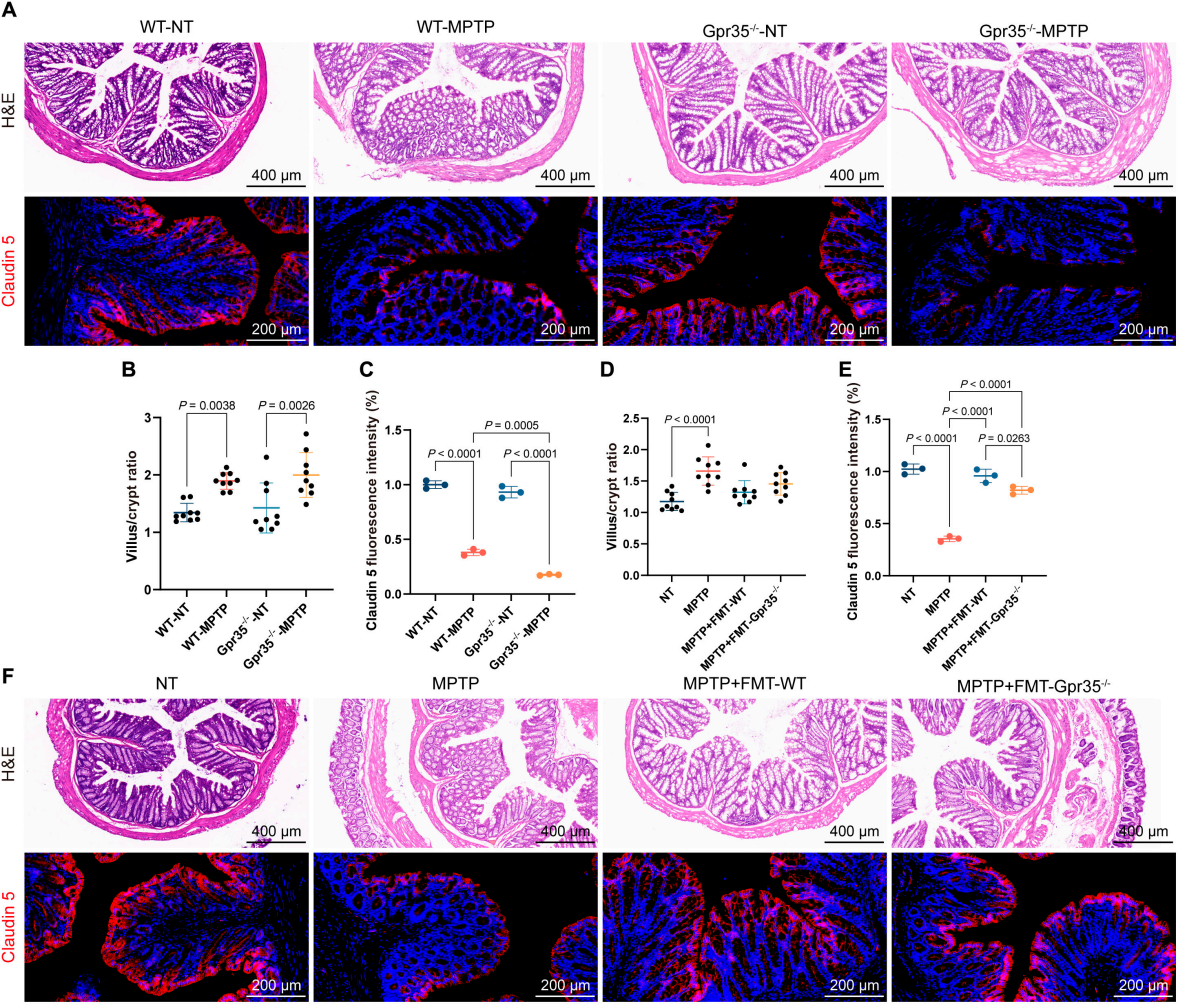
Effects of Gpr35 on intestinal structure and barrier integrity in PD mice. (A and F) Representative images of hematoxylin and eosin (H&E) staining (top) and fluorescence staining of Claudin 5 (bottom) in the colon. (B and D) Quantitative analysis of villus length and crypt depth (*n* = 5 biologically independent mice). (C and E) Quantification of Claudin 5 fluorescence intensity (*n* = 3 biologically independent mice).

We have demonstrated that Gpr35^−/−^ exacerbated PD progression and inflammation, and this effect was mediated by alterations in intestinal flora. Considering the predominant expression of Gpr35 in the gut epithelium, we further explored the effects of Gpr35^−/−^ on the gut. In WT-MPTP mice, colitis-like pathological features were observed, including an increased villus-to-crypt length ratio (Fig. 4B), abnormal morphology of intestinal mucosal crypts, and edema, although no inflammatory cell infiltration was detected compared to WT-NT mice (Fig. 4A, top). In Gpr35^−/−^-MPTP mice, the villus-to-crypt length ratio was significantly elevated (Fig. 4B), alongside inflammatory cell infiltration and abnormal crypt architecture in the intestinal mucosa compared to Gpr35^−/−^-NT mice (Fig. 4A, top). FMT experiments further demonstrated that microbiota from WT mice alleviated edema and inflammatory cell infiltration in PD mice. However, PD mice receiving microbiota from Gpr35^−/−^ mice continued to exhibit intestinal edema and inflammatory cell infiltration (Fig. 4F, top). We also analyzed intestinal barrier integrity by staining for Claudin 5. Claudin 5 expression was reduced in both WT-MPTP and Gpr35^−/−^-MPTP mice after MPTP induction (Fig. 4A, bottom, and C). However, Gpr35^−/−^-MPTP mice exhibited more severe barrier integrity damage compared to WT-MPTP mice (Fig. 4A, bottom, and C). In FMT-treated mice, intestinal barrier integrity was restored to varying degrees by flora from both WT and Gpr35^−/−^ mice, with WT flora demonstrating significantly better restorative effects (Fig. 4F, bottom, and E). Notably, in the FMT experiments, microbiota transplantation from either WT or Gpr35^−/−^ mice failed to restore the villus/crypt ratio, demonstrating that while FMT could not reverse PD-induced structural alterations in intestinal morphology, it effectively ameliorated both intestinal inflammation and barrier integrity impairment caused by PD. In conclusion, Gpr35 stabilizes intestinal structure and barrier integrity in both MPTP-induced and FMT-treated mouse models, further underscoring the connection between Gpr35, gut flora, and PD pathology.

### Gpr35 agonist KYNA inhibits neuronal apoptosis and microglial inflammatory responses

**Fig. 5.**
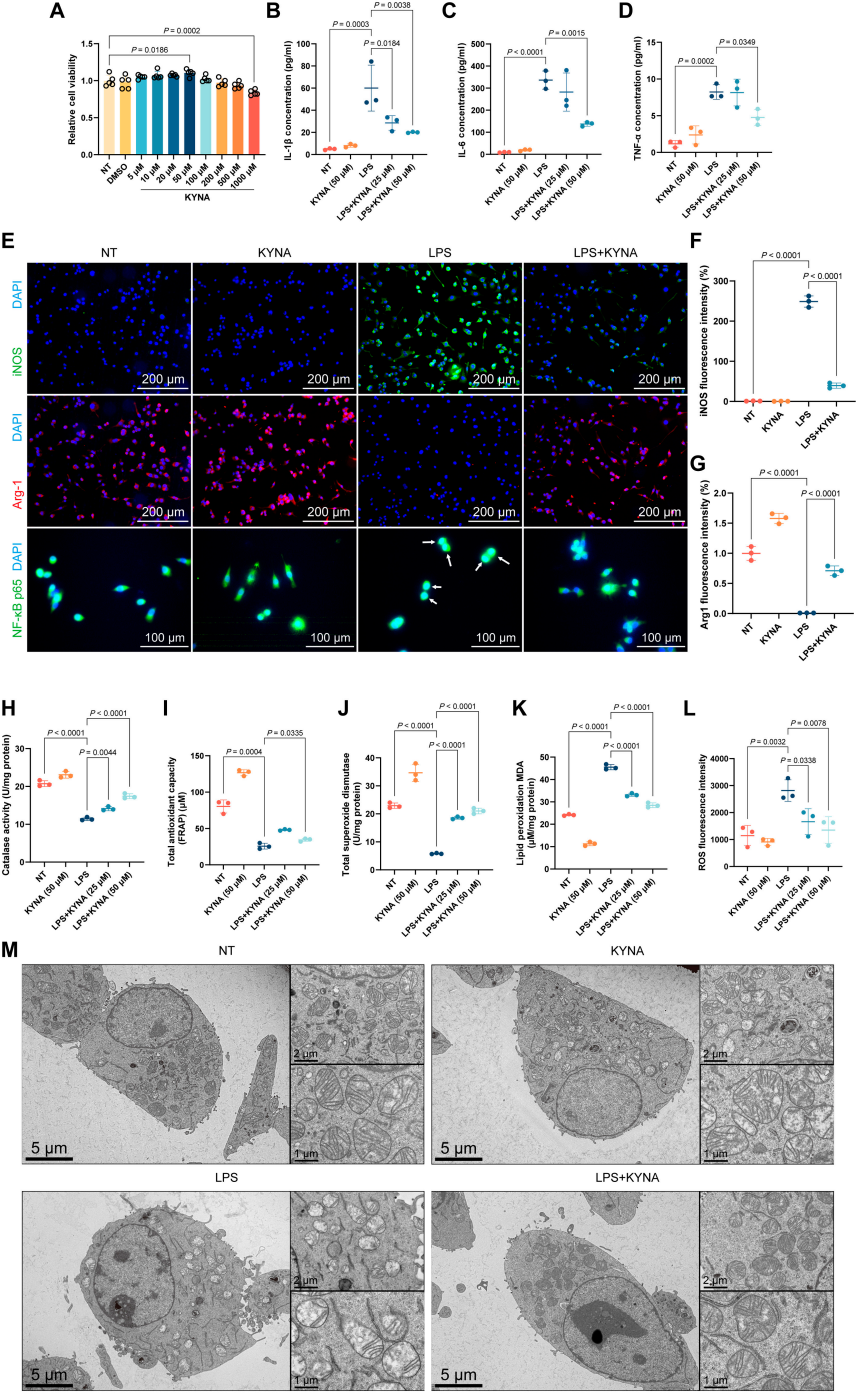
Modulation of microglial activation and neuroprotective effects by Gpr35 agonist KYNA in murine cellular models. (A) Concentration-dependent effects of KYNA (5 to 1,000 μM) on BV-2 microglial cell viability assessed by CCK-8 assay. (B to D) KYNA-mediated suppression of proinflammatory cytokine secretion in BV-2 cells quantified by enzyme-linked immunosorbent assay (ELISA): interleukin-1β (IL-1β; B), interleukin-6 (IL-6; C), and tumor necrosis factor-α (TNF-α; D). (E) Immunofluorescence visualization of polarization markers in BV-2 cells: iNOS, M1 marker (upper panel); Arg-1, M2 marker (middle panel); and NF-κB p65 subunit translocation (lower panel). Scale bar, 20 μm. (F and G) Semiquantitative analysis of iNOS (F) and Arg-1 (G) fluorescence intensity normalized to 4′,6-diamidino-2-phenylindole (DAPI) nuclear staining. (H to L) Comprehensive evaluation of KYNA’s antioxidant effects in BV-2 cells. (H) CAT enzymatic activity. (I) Total antioxidant capacity assessed by ferric reducing antioxidant power (FRAP) assay. (J) SOD activity. (K) MDA content reflecting lipid peroxidation. (L) Intracellular ROS levels detected by dichloro-dihydro-fluorescein diacetate (DCFH-DA) fluorescence. (M) Ultrastructural analysis of mitochondrial integrity by TEM. Yellow arrowheads indicate cristae morphology. Scale bar, 500 nm. Data represent mean ± SEM from ≥3 biological replicates. Statistical significance was determined by one-way ANOVA with Tukey’s post hoc test.

To explore the effect of Gpr35 expression on PD in brain, we established an in vitro neuroinflammation model by stimulating BV-2 microglial cells with lipopolysaccharide (LPS) and an in vitro neuronal apoptosis model by treating SN4741 dopaminergic neurons with 1-methyl-4-phenylpyridiniumion (MPP^+^). The potential cytotoxic effects of KYNA on BV-2 microglia were evaluated through the Cell Counting Kit-8 (CCK-8) assay. Results indicated that 50 μM KYNA significantly enhanced microglial cell viability, whereas 1,000 μM KYNA significantly inhibited cell activity (Fig. 5A). Additionally, KYNA significantly inhibited the release of inflammatory cytokines induced by LPS (Fig. 5B to D). As a critical regulator of inflammatory responses, nuclear factor κB (NF-κB) functions as a key transcriptional modulator. After KYNA treatment, p65 was primarily found in the cytoplasm, thereby partially terminating the signaling of pro-inflammatory factors (Fig. 5E). At the protein level, KYNA was observed to significantly inhibit the expression of cyclooxygenase-2 (COX-2) and iNOS. Further, it promoted the expression of the anti-inflammatory protein Arginase-1 (Arg-1; Fig. 5E to G and Fig. S5E to G). Given that oxidative stress can exacerbate inflammatory responses and contribute to neurodegenerative and metabolic diseases, the total antioxidant capacity, nicotinamide adenine dinucleotide/nicotinamide adenine dinucleotide (reduced form) (NAD^+^/NADH) ratio, and levels of glutathione (GSH), catalase (CAT), and superoxide dismutase (SOD) in BV-2 cells were measured. Results showed that LPS treatment reduced the oxidation resistance of BV-2 cells, an effect that was effectively reversed by KYNA treatment (Fig. 5H to J and Fig. S5C and D). Additionally, KYNA efficiently cleared the accumulation of harmful oxidative products, such as reactive oxygen species (ROS) and malondialdehyde (MDA), which are induced by inflammation (Fig. 5K and L). Transmission electron microscopy (TEM) revealed that LPS-treated BV-2 cells exhibited marked mitochondrial damage compared to the control group, characterized by sparse and fragmented mitochondrial cristae. Some mitochondria changed from a normal oval shape to a swollen, rounded morphology, suggesting mitochondrial permeability transition and outer membrane rupture. However, KYNA treatment partially restored these abnormal mitochondrial morphologies (Fig. 5M). JC-1 staining further confirmed that KYNA effectively alleviated the LPS-induced decline in mitochondrial membrane potential (Fig. S5A and B). Collectively, these results indicate that KYNA, acting as a putative Gpr35 agonist, regulates inflammatory responses and reduces oxidative stress, effectively mitigating mitochondrial structural impairment and membrane potential decline. By maintaining mitochondrial integrity in BV-2 cells, KYNA may contribute to slowing PD progression.

### KYNA reprograms multi-omics profiles in BV-2 microglia

To elucidate molecular-level alterations and their regulatory mechanisms, we performed integrated transcriptomic and proteomic profiling of microglia treated with Gpr35 agonists. Comparative analysis across experimental groups quantified differentially expressed genes (DEGs) and proteins (DEPs).

**Fig. 6.**
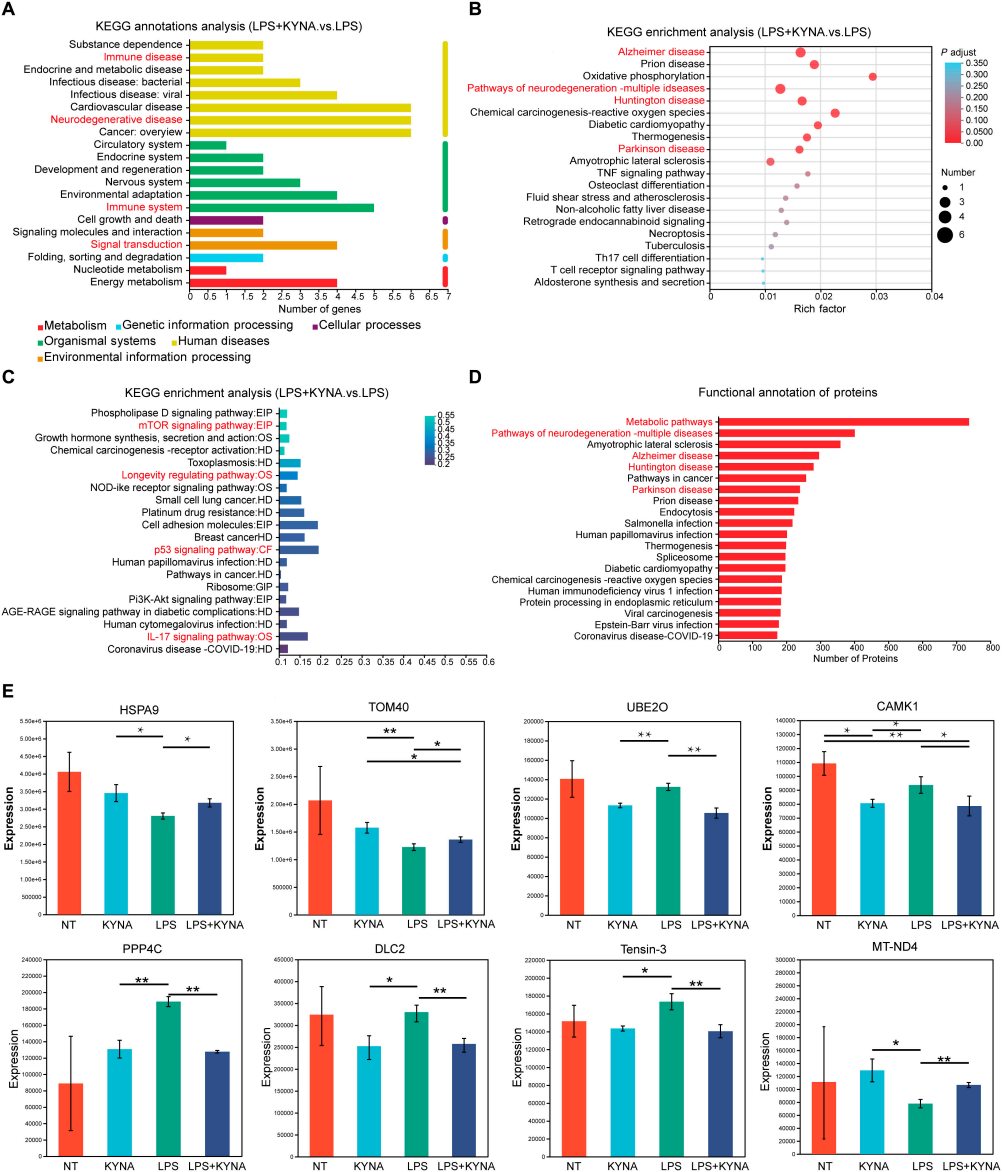
Transcriptomic and proteomic profiling of BV-2 cells in response to Gpr35 agonist KYNA treatment. (A) KEGG functional annotation of DEGs between the LPS-treated and KYNA + LPS-treated groups. (B) KEGG pathway enrichment analysis of DEGs, highlighting functional differences between the LPS and KYNA + LPS groups. (C) KEGG pathway enrichment analysis of DEPs, highlighting functional differences between the LPS and KYNA + LPS groups. (D) Functional classification histogram of proteins based on EggNOG database. (E) Expression levels of representative DEPs (raw intensity values) between LPS and KYNA + LPS groups. Differential proteins were identified based on FC criteria (FC > 1.2 or FC < 0.833 with *P* < 0.05), but raw expression intensities are displayed to visualize absolute abundance changes. Data are presented as mean ± SEM; ^*^P < 0.05, ^**^P < 0.01.

KEGG annotation revealed that DEGs between LPS-treated and KYNA + LPS-treated groups were predominantly associated with neurodegenerative diseases, signal transduction cascades, and immune system regulation (Fig. 6A). Functional enrichment analysis revealed marked associations of the DEGs with major neurodegenerative disorders [PD, Huntington’s disease, and Alzheimer’s disease (AD)], potentially mediated through impaired oxidative phosphorylation and aberrant tumor necrosis factor (TNF) signaling. These findings were corroborated by parallel protein functional annotation results (Fig. 6B and D).

Parallel proteomic quantification identified marked DEPs across experimental groups (Fig. S6C and D). The Subloc annotation demonstrates the spatial localization of these proteins (Fig. S6E), with the functional annotation highlighting their roles in metabolic reprogramming and neurodegenerative disease-related pathways (Fig. 6D). KEGG enrichment analysis of DEPs between the 2 groups found that these markedly different proteins are primarily associated with the mTOR (mammalian target of rapamycin) signaling pathway, cellular senescence regulation, and IL-17 and p53 signaling pathway (Fig. 6C). Subcellular localization localized these DEPs primarily to the cytoplasm, nucleus, and mitochondria (Fig. S6F).

LPS treatment was found to significantly down-regulate mitochondrial outer membrane transport proteins, including the outer mitochondrial membrane 40 (TOM40) and heat shock protein A9 (HSPA9), as well as NADH dehydrogenase 4 (MT-ND4), a critical subunit of mitochondrial respiratory chain complex I. Additionally, LPS activated several key signaling molecules: calcium/calmodulin-dependent protein kinase 1 (CAMK1), which is associated with calcium channel regulation; ubiquitin-conjugating enzyme E2D4 (UBE2O), an E2 ubiquitin-conjugating enzyme implicated in apoptosis; protein phosphatase 4 catalytic subunit (PPP4C), a phosphatase involved in cell cycle progression; and cancer-related proteins deleted in liver cancer 2 (DLC2) and tensin-3 (Fig. 6E). Importantly, administration of KYNA effectively reversed these LPS-induced alterations, restoring mitochondrial transport protein expression levels and attenuating pro-apoptotic signaling pathways. This suggests that activation of Gpr35 alleviates inflammation-mediated mitochondrial dysfunction and associated cellular stress.

### Gpr35 small interfering RNA knockdown reverses KYNA-mediated anti-inflammatory, antioxidant, and neuroprotective effects

**Fig. 7.**
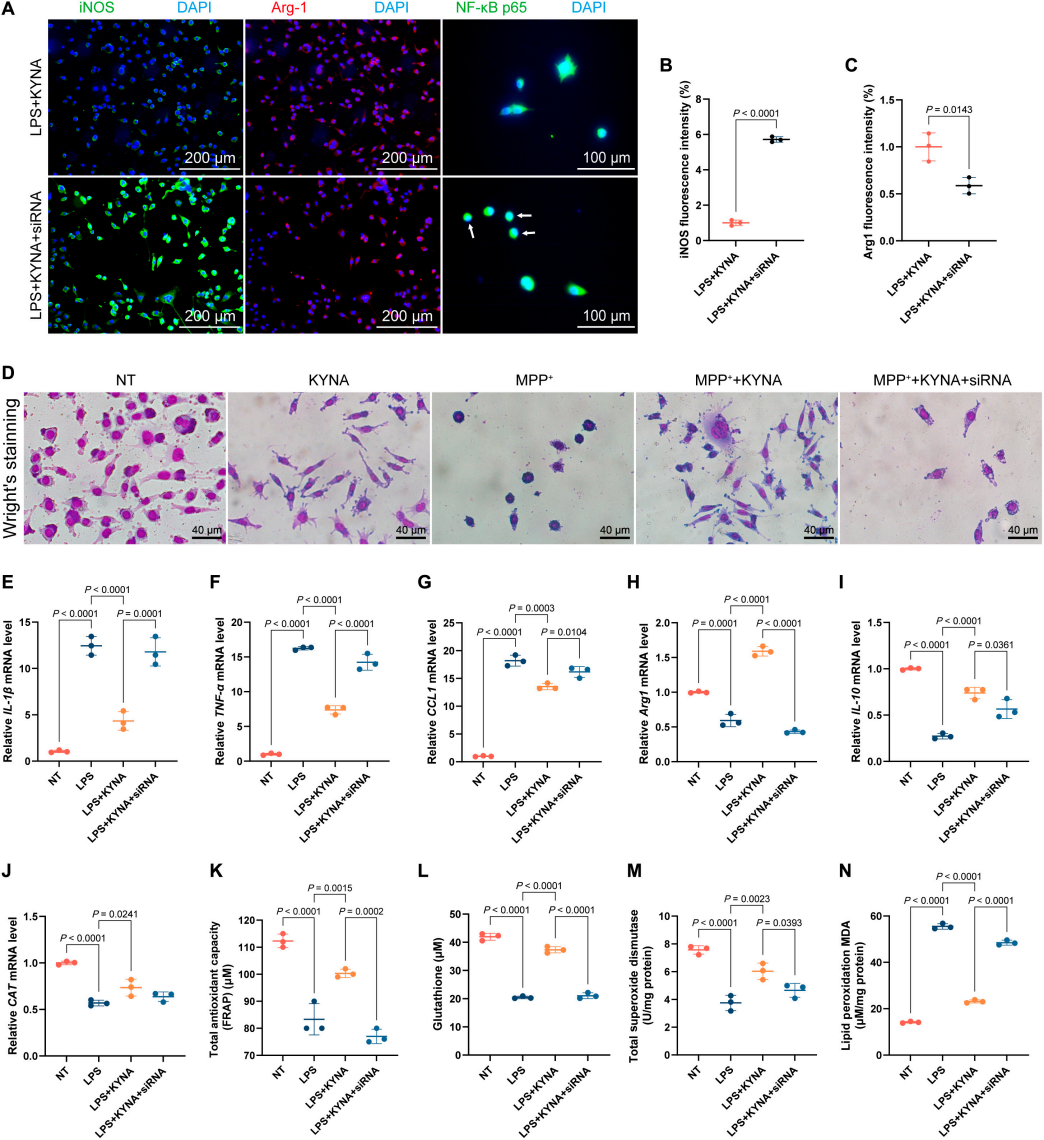
Impact of Gpr35 siRNA knockdown on BV-2 and SN4741 cells. (A) Representative immunofluorescence images showing the expression of iNOS (left), Arg-1 (middle), and NF-κB p65 (right) in BV-2 microglial cells with or without Gpr35 siRNA knockdown. (B and C) Quantification of relative fluorescence intensity for iNOS (B) and Arg-1 (C) from immunofluorescence staining. (D) Wright’s staining of SN4741 cells following MPP^+^ stimulation and treatment under different conditions. (E to J) RNA expression of inflammatory cytokines (*IL-1β*, *TNF-α*, *CCL1*, *Arg-1*, *IL-10*, and *CAT*) in BV-2 microglial cells. (K to N) Analysis of oxidative stress markers in BV-2 cells: total antioxidant capacity measured by the FRAP assay (K), GSH levels (L), total SOD concentration (M), and MDA levels (N). Data are pooled from at least 2 independent experiments and presented as mean ± SEM.

To further investigate whether Gpr35 is a key protein mediating KYNA regulation of inflammation, oxidative stress, and apoptosis, we constructed small interfering RNA (siRNA) targeting Gpr35. To identify the most effective siRNA candidate, we evaluated the silencing efficiency of 3 Gpr35-targeting siRNAs (provided by Genema Biotech) in both BV-2 and SN4741 cell lines using reverse transcription quantitative polymerase chain reaction (RT-qPCR) analysis. As demonstrated in Fig. S7, siRNA-3 exhibited significantly superior knockdown efficiency compared to the other 2 candidates. Our results demonstrated that KYNA promotes M2 polarization (alternative activation) while inhibiting M1 polarization (classical activation) in BV-2 microglial cells. However, when cells were treated with siRNA targeting Gpr35, the effects of KYNA were significantly reversed (Fig. 7E to I and Fig. S8D to K).

Additionally, siRNA treatment reversed the regulatory effects of KYNA on cellular antioxidant capacity [*glutamate-cysteine ligase catalytic subunit* (*GCLC*), g*lutamate-cysteine ligase modifier subunit* (*GCLM*) (Fig. S8L and M), and *CAT* (Fig. 7J)] and also reversed the expression of GSH and SOD (Fig. 7L and M). Consequently, harmful oxidative species accumulated excessively within the cells (Fig. S6N). In the neuronal apoptosis model, The CCK-8 viability analysis indicated that MPP^+^ induced a concentration-dependent inhibition of cell viability (Fig. S5H), which was also confirmed by the lactate dehydrogenase (LDH) assay results (Fig. S5I). Meanwhile, the siRNA diminished the protective effects of KYNA on SN4741 cell viability (Fig. S5K). Similarly, in the coculture system of BV-2 and SN4741 cells, siRNA inhibited the protective effects of KYNA on SN4741 cells (Fig. S5J), as confirmed by fluorescence staining of the apoptotic marker Caspase 3 (Fig. S8B and C). Furthermore, Wright and Nissl staining showed that KYNA improved the morphological changes induced by MPP^+^ in neurons, such as reduced synaptic density, rounding of the cell body, and a marked decrease in cell density. However, siRNA treatment reversed these effects (Fig. 7D and Fig. S8A).

In summary, while siRNA-mediated reversal of KYNA’s effects suggests Gpr35 as a potential regulatory target, the absence of negative controls in the experiments warrants cautious interpretation of these findings. In conclusion, Gpr35 not only delays PD progression by modulating gut microbiota and metabolites but also directly regulates PD pathogenesis in the nervous system by controlling inflammation, oxidative stress, and neuronal apoptosis.

## Discussion

The course and development of PD are related to neuroinflammation, intestinal microbial disorders, neuronal apoptosis, and oxidative stress [26]. Studies indicate that microglia in the SN of PD patients are chronically activated, leading to increased levels of neuroinflammatio [27]. Under inflammatory conditions, activated microglia release pro-inflammatory mediators, leading to dopaminergic neuron damage [28]. Mounting evidence underscores the pivotal involvement of intestinal microbial communities and their metabolic byproducts in PD pathogenesis [8,9]. It is worth noting that previous studies failed to identify therapeutic or intervention targets in line with the functional principle of gut–brain association.

Gpr35 has been reported to be capable of regulating intestinal barrier integrity and brain neuroplasticity [29,30], but it failed to be unveiled as the essential role in bridging the gut and brain association or neurodegenerative symptoms. Our investigation provides novel insights into Gpr35’s neuroprotective function in PD, revealing that genetic ablation of Gpr35 aggravates disease pathology through enhanced motor deficits, accelerated dopaminergic neuron degeneration, and amplified neuroinflammatory responses in PD mouse models. Through FMT and multi-omics analysis, we reveal that Gpr35 modulates PD pathogenesis by regulating the gut microbiota, particularly lactic acid bacteria, and metabolites related to neurotransmitters and lipid metabolism. Furthermore, the Gpr35 agonist KYNA exhibits marked neuroprotective, anti-inflammatory, and antioxidant effects in vitro, while siRNA targeting Gpr35 suppresses these effects, as demonstrated using microglia and neuron cell models. Integrated gut–brain evidence suggests that Gpr35 may function through a “dual gut–brain mechanism”, which explains the therapeutic effects observed with both FMT and KYNA treatment.

This research conducted a systematic exploration of Gpr35’s impact on PD progression, focusing specifically on its relationship with intestinal microbial communities and associated metabolic products. Our findings revealed that Gpr35 deficiency resulted in a reduction of *Lactobacillus* populations within the gut microbiome, a phenomenon that becomes more pronounced during PD progression. Contemporary scientific literature emphasizes the crucial involvement of intestinal flora and their metabolic byproducts in PD pathogenesis [31,32]. This might be attributed to 2 main factors: First, PD can lead to intestinal disease due to autonomic dysfunction [33]; additionally, alterations in microbial composition and metabolite profiles directly affect neuroinflammatory processes underlying disease progression [34]. For instance, *Prevotella* and *Lactobacillus* are significantly reduced in PD patients [35]. Probiotic supplementation, particularly with *Lactobacillus*, has been shown to alleviate constipation and potentially improve motor symptoms by modulating gut microbiota [36]. Clinical evidence confirms that multi-strain probiotic formulations containing *Lactobacillus* enhance intestinal motility and reduce transit time in PD patients with constipation [37], findings that align with our study. Multi-omics analyses and colonization experiments identify *Lactobacillus* as a key microbiota mediator of Gpr35’s regulatory effects in PD, although future validation using genetically engineered strains is required to establish causality.

Gut microbiota regulation of host physiology is closely linked to intestinal metabolites [38]. In WT and Gpr35^−/−^ PD mice, our analysis revealed marked enrichment in several neural signaling pathways, including neuroactive ligand–receptor interactions, along with tyrosine and tryptophan metabolic processes. The enzymatic activity of aromatic amino acid aminotransferase (ArAT) and indole lactate dehydrogenase (ILDH) enables *Lactobacillus* strains to metabolize tryptophan into indole derivatives including indolyl aldehyde and indolyl lactic acid [39]. These metabolites activate aromatic receptors (AHRs), which regulate intestinal immune barrier function [40], suppress inflammation, and enhance gut barrier integrity. *Lactobacillus* may also regulate tyrosine metabolism [41], influencing neurotransmitters like dopamine and norepinephrine, which affect neurobehavior via the gut–brain axis [42]. Additionally, vitamin B2, KYNA, and trigonelline, which are related to these metabolic pathways [43–45], were also enriched in our study. The accumulation of KYNA following Gpr35 gene knockout may reflects a compensatory elevation of ligand levels in response to impaired receptor signaling, which deserves further research in the future. While our metabolomics analysis revealed marked alterations in tryptophan/tyrosine metabolic pathways, the functional implications remain to be validated through in vitro isotope tracing or in vivo metabolic flux analysis. Future studies employing stable isotope labeling (e.g., ^13^C-tryptophan) or mono-colonization models will elucidate the direct effects of *Lactobacillus*-derived metabolites on neuroinflammation.

For examination of Gpr35’s contribution to PD mechanisms within the brain, we explored the cerebral environment in PD mice. Literature indicates that PD progression exacerbates neuroinflammation, oxidative stress, and neuronal apoptosis in the affected regions [46]. KYNA has been found in some studies that exerts anti-inflammatory effects via the Gpr35 protein in neuroinflammation and pneumonia [23,47]. KYNA also significantly up-regulates antioxidant enzyme expression in the ovine ventricle [48]. Furthermore, KYNA specifically inhibits NLRP3 inflammasome activation through Gpr35, thereby preventing Caspase 1 cleavage [49]. In our study, we found that activating Gpr35 inhibits BV-2 cell activation by suppressing NF-κB p65 nuclear translocation and promotes M2 polarization. This activation also enhances the antioxidant capacity of BV-2 cells. In SN4741 cells, Gpr35 activation effectively inhibits neuronal apoptosis by suppressing Caspase 3 expression. Our study found that targeting Gpr35 can effectively regulate PD.

Although the final validation experiments lacked scramble controls, we had previously confirmed that siRNA-3 achieved significantly higher knockdown efficiency than negative controls (NC) during preliminary screening. Moreover, this specific sequence has been functionally validated in prior studies [50,51]. While these siRNA results should be considered preliminary evidence, we plan to further verify target specificity by generating Gpr35 conditional knockout cell lines or employing CRISPR-based approaches in future investigations. Our study acknowledges certain limitations in experimental modeling and mechanistic exploration. The MPTP-induced PD model primarily mimics acute neurotoxicity rather than the chronic, multifactorial nature of human PD, and it fails to replicate α-syn aggregation [52]. Furthermore, the current model demonstrates limitations in replicating nonmotor symptoms [35], which represents an area requiring further refinement in future investigations, This may lead to an incomplete understanding of PD pathogenesis and affect the study of Gpr35 mechanisms. Nevertheless, MPTP exhibits selective neurotoxicity specifically targeting the nigrostriatal dopaminergic pathway, which closely recapitulates the cardinal pathological hallmark of PD while demonstrating minimal effects on other neuronal populations, coupled with its advantages of producing limited systemic toxicity in animals and high experimental reproducibility [53], thereby establishing it as an indispensable tool for PD research. The current study did not employ conditional knockout models (e.g., Villin-Cre or Cx3cr1-Cre), thus precluding definitive discrimination between Gpr35’s independent roles in intestinal epithelial versus microglial cells. All references to “dual mechanisms” or “gut–CNS bidirectional regulation” in this work should be interpreted as speculative hypotheses rather than causal conclusions. Future investigations utilizing cell type-specific knockout models will be required for definitive validation. For instance, downstream pathways of Gpr35 in regulating gut microbiota and neuroinflammation are not fully defined, and KYNA’s impact on signaling pathways like NF-κB requires further validation. Beyond the 2 Gpr35-mediated regulatory pathways in PD pathogenesis elucidated in this study, its well-documented role in peripheral immune modulation may represent an additional mechanistic pathway influencing PD progression, which warrants systematic investigation in future research [19,54]. Additionally, KYNA, as a Gpr35 agonist, may interact with other receptors such as N-methyl-D-aspartate receptor (NMDAR) [55], and whether its anti-inflammatory effects in vitro fully depend on Gpr35 needs to be verified with more specific tools.

To address these limitations, future research should employ organoid models and clinical trial data that better mimic human characteristics. Human-derived gut–brain axis organoid models could simulate how microbial metabolites (e.g., KYNA) affect neuroinflammation via Gpr35. Furthermore, as an endogenous agonist of Gpr35, KYNA has been systematically evaluated in cognitive disorders through a comprehensive review and meta-analysis [56]. Future clinical drug development for PD should rigorously assess the blood–brain barrier permeability and chronic toxicity profiles of KYNA analogs. We propose implementing combination strategies, such as co-administration of KYNA with *Lactobacillus*, to enhance therapeutic efficacy and facilitate clinical translation. Integrating multi-omics data analysis may also offer a more comprehensive view of PD pathogenesis and help establish the brain–gut–metabolism regulatory axis through potential correlations among multiple indicators.

## Conclusion

**Fig. 8.**
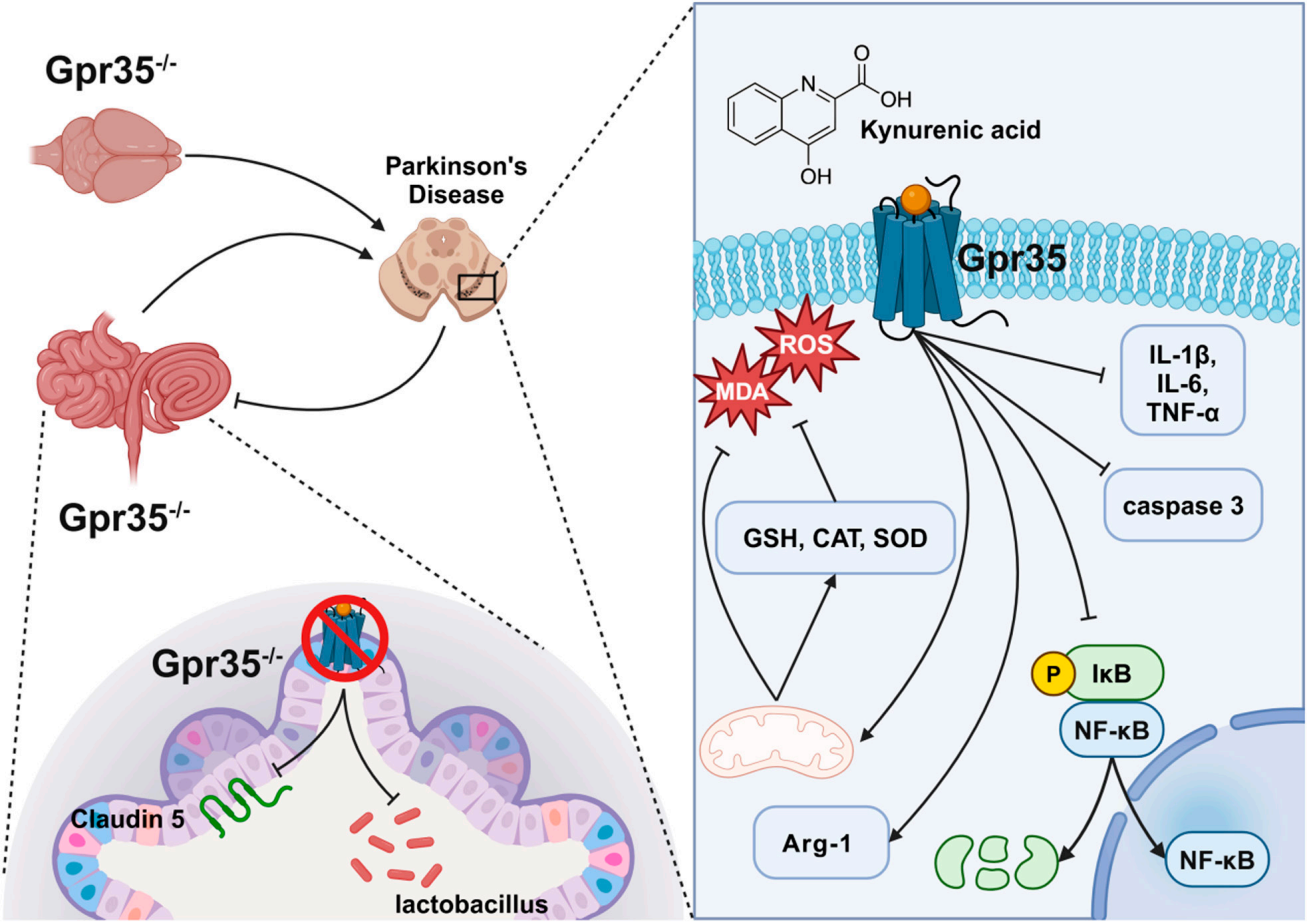
Schematic summary of the study. Gpr35 signaling attenuates neuroinflammation, promotes enrichment of gut Lactobacillus, and ameliorates motor deficits in the MPTP mouse model of PD.

As shown in Figure 8, this study systematically explored the role of Gpr35 in PD and its regulatory mechanisms. This study systematically explored the role of Gpr35 in PD and its regulatory mechanisms. Gpr35 deficiency was found to exacerbate motor dysfunction, dopaminergic neurodegeneration, and neuroinflammation in PD models, while activation of Gpr35 by the agonist KYNA demonstrated substantial neuroprotective and anti-inflammatory effects. Additionally, Gpr35 modulates PD pathology by influencing gut microbiota composition (particularly *Lactobacillus*) and metabolic pathways such as tryptophan and tyrosine metabolism. These findings preliminarily suggest that Gpr35 may participate in PD progression through dual pathways involving gut microbiota and neuroinflammation, providing a theoretical foundation for subsequent mechanistic investigations. However, the causal relationships require further validation through cell-specific models and functional microbiota studies.

## Materials and Methods

### Chemicals and reagents

This section is presented in Table S1.

### Mice

All experimental protocols involving animals were carried out in compliance with ethical standards for laboratory animal welfare, with official authorization granted by the Institutional Animal Care and Use Committee at Jilin University (authorization code: SY202411002).

Male C57BL/6J mice (specific pathogen-free, aged 8 weeks) were acquired from Benxi-based Changsheng Biotechnology Co. Ltd. (China). Gpr35-deficient mice (model number: NM-KO-2106644) were sourced from Shanghai Model Organisms Center Inc. (Shanghai, China). The animals were housed in standard laboratory facilities maintained under controlled environmental conditions. These facilities operated on a 12-h photoperiod cycle with ambient temperature regulation at 22 ± 2 °C. Throughout the study period, all subjects received unrestricted nutritional provisions and hydration access.

For the animal experiments, while key assays (immunofluorescence quantification and RT-qPCR) were performed with a sample size of *n* = 3—meeting the minimum requirements for preliminary exploratory studies—this limited scale was constrained by available resources and time constraints. Consequently, these histological and molecular data should be interpreted as supportive evidence, with primary conclusions drawn from the larger-scale behavioral tests (*n* = 8 to 10). Future replication studies with expanded sample sizes are warranted to validate these findings.

### Subacute MPTP model induction and FMT administration

After adaptation for 1 week, the mice were induced with a PD model via a subacute method as previously described [52]. The experimental protocol involved administering daily intraperitoneal doses of MPTP [99.54% purity, dissolved in phosphate-buffered saline (PBS)] at 30 mg/kg/day over 7 consecutive days. Model validation assessments were conducted 7 d after completing the treatment regimen.

For FMT experiments, the intrinsic microbiota of the mice was cleared by administering an antibiotic solution consisting of penicillin (1 g/l), metronidazole (1 g/l), neomycin (1 g/l), and vancomycin (0.5 g/l) in their drinking water over a 14-d period. During the first week, antibiotics were also administered orally (penicillin, 200 mg/kg; metronidazole, 200 mg/kg; neomycin, 200 mg/kg; vancomycin, 100 mg/kg). For the subsequent week, only antibiotic-containing drinking water was provided to the mice to maintain microbiota depletion. In this period, we also monitored the weight changes and water intake. Fresh feces were collected from control and Gpr35^−/−^ mice, and we placed them in a sterile container, with 600 mg of samples rapidly homogenized in 6 ml of saline for 60 s. This mixture underwent filtration through sterile mesh to eliminate particulate matter before centrifugation at 1,000*g* (4 °C) for 3 min. The resulting supernatant was used for oral inoculation of recipient mice within 10 min using gastric gavage. Post-transplantation monitoring included fecal sample collection within 7 d for microbial analysis through either 16*S* rRNA sequencing or bacterial culture techniques. We aliquoted the unused fecal suspension and added 20% sterile glycerol before storing it at −80 °C in an ultra-low temperature freezer. The storage duration was limited to no more than 6 months to ensure sample integrity.

### *Lactobacillus* colonization

We revived the cryopreserved *Lactobacillus* strains by selecting a small number of colonies and inoculating them into Man Rogosa Sharpe broth (MRS) liquid medium. Cultivation occurred under static conditions at 37 °C for 18 to 24 h, with periodic optical density measurements tracking microbial proliferation. Once turbidity was evident, we harvested a small aliquot of the culture for Gram staining and microscopic examination to confirm the morphological integrity and purity of the *Lactobacillus* strains. Subsequent bacterial separation involved centrifugation at 4,000*g* for 10 min, followed by triple washing cycles using physiological saline to eliminate medium residues. The cellular precipitate was reconstituted in sterile saline solution, with viable counts quantified through serial dilution plating techniques. Daily oral administration of bacterial suspensions (10^7^ to 10^9^ viable units per animal) continued for 3 weeks. MPTP-induced Parkinsonian models were established through intraperitoneal neurotoxin injection on day 14 of the regimen. One week after PD induction, we evaluated the effects of *Lactobacillus* on PD mice through a series of behavioral experiments.

### Behavioral test

Behavioral experiments were conducted 1 week after the completion of drug administration.

For the open-field experiment [57], a rectangular enclosure with 25- to 30-cm wall height and quadrilateral floor dimensions of 72 cm per side was employed. Black paint was applied to the interior surfaces to maintain consistent visual contrast. A digital camera was mounted 2 m above the apparatus to ensure complete and clear coverage of the entire open field. The test was conducted in a quiet, well-lit room to minimize external disturbances. We placed each mouse in the center of the box and simultaneously initiated video recording and timing. The mice freely explored the environment for a 300-s observation period before termination of video capture. Comprehensive sanitization protocols involving 70% ethanol solution were implemented between trials to eliminate olfactory cues from previous subjects, with particular attention given to flooring and vertical surfaces. This procedure was repeated for each mouse. After completing the recordings, we used the AnyMaze software to analyze several key parameters: central zone occupancy duration, ambulatory path length summation, and movement trajectory of the mice over time. These quantitative measurements facilitated comparative evaluation of locomotor activity patterns and anxiety-related behavioral modifications during novel environment exploration.

For the pole descent assessment, the protocol outlined by Ogawa et al. [58] was implemented with minor modifications. A vertically positioned 50-cm-high plastic rod featuring a coarse surface texture (1.5 mm diameter) served as the climbing apparatus. Experimental animals were gently placed at the structure’s apex in a head-down orientation, with descent duration to the base recorded as the primary measurement. Each subject underwent 3 consecutive trials with an intertrial rest period of 1 min. Subsequent data processing involved computing the mean value from all attempts for analytical purposes, ensuring consistent evaluation metrics across test subjects.

### Preparation of histological samples

Animals were heavily anesthetized via intraperitoneal administration of pentobarbital solution (100 mg/kg). A thoracotomy was performed to access the cardiac region, where a perfusion needle was introduced into the left ventricular chamber. Initial perfusion with approximately 20 ml of physiological saline effectively cleared circulatory blood, succeeded by infusion of 50 ml of 0.1 M PBS containing 4% (w/v) paraformaldehyde for tissue fixation. Following craniotomy, the complete cerebral structure was meticulously dissected and transferred to a 4% paraformaldehyde solution for extended post-fixation. Fixed specimens underwent 4 °C storage for 6 to 24 h before immersion in 30% sucrose solution until tissue sedimentation occurred under refrigerated conditions. Cryopreserved samples were embedded in optimal cutting temperature (OCT) medium, with subsequent generation of 40-μm coronal sections using cryosectioning technology. Sectioned tissues underwent triple washing cycles in 0.01 M PBS solution prior to histological processing.

### Immunofluorescence staining

For immunofluorescence analysis, tissue sections were initially exposed to 0.01 M PBS supplemented with 1% caprine serum for 40 to 60 min at ambient temperature to prevent unspecific interactions. After blocking, the sections were incubated overnight at 4 °C with the primary antibody mixture diluted in antibody diluent. Post-primary antibody treatment, thorough rinsing with 0.01 M PBS preceded transfer to light-protected secondary antibody solution for 2 to 5 h of ambient temperature incubation. Following final PBS washing cycles, samples were carefully positioned on precleaned microscopy slides under low-light conditions. Complete desiccation preceded application of fluorescence-compatible sealant medium incorporating photobleaching inhibitors (conventional mounting medium for nonfluorescent applications), with subsequent dark storage at 4 °C until microscopic examination.

### Nissl staining

For Nissl staining, brain sections obtained from mice were exposed to tar violet solution through incubation at 56 °C for 1 h. Following this process, the slides were rinsed with deionized water to remove excess stain. We then briefly treated the slides with a differentiation solution, monitoring under a light microscope until the background appeared transparent. Sequential dehydration procedures were conducted employing pure ethanol solutions and xylene immersion. Tissue transparency was enhanced through additional xylene treatment before final preservation. The processed sections were permanently sealed using neutral balsam mounting medium for microscopic examination.

### Hematoxylin and eosin staining

The tissue samples were immersed in hematoxylin solution for variable durations between 2 and 10 min based on staining requirements. Following hematoxylin application, excess stain was eliminated through multiple distilled water washes. Acidic alcohol solution was subsequently applied for controlled destaining. Specimens then underwent counterstaining in eosin solution for 1- to 5-min intervals before thorough rinsing with purified water. Sequential dehydration was achieved by processing through ascending ethanol concentrations. Final preparation involved immersion in a clearing agent for microscopic evaluation. Throughout the procedure, staining durations were precisely monitored and adjusted according to tissue characteristics.

### Wright staining

The Wright stain was formulated by mixing suitable quantities of eosin Y and methylene blue dye in methanol solvent. For cell smear preparations, a few drops of Wright staining solution were applied to cover the entire smear, and the slides were stained for approximately 1 min. An equivalent quantity of PBS was subsequently added to dilute the staining mixture, followed by maintaining the slides undisturbed for 5 to 10 min under ambient temperature conditions. Post-staining procedures involved rigorous rinsing under flowing tap water to remove excess dye, subsequent natural drying of specimens, and final microscopic evaluation.

### 16*S* rRNA sequencing and data analysis

The experimental procedures, including sample collection, DNA extraction, amplicon design, PCR amplification, and sequencing, were conducted by Novogene in Beijing, China. Specifically, the microbial community’s total genomic DNA was isolated, with its integrity verified through 1% agarose gel electrophoresis. Quantitative evaluation of DNA concentration and purity measurements were executed using a NanoDrop 2000 spectrophotometer (Thermo Fisher Scientific, USA). Amplification of the 16*S* rRNA gene’s v3-v4 hypervariable regions was achieved through PCR amplification, employing the isolated DNA as the template with specific primers: the forward primer *338F* (5′-ACTCCTACGGAGGCAGCAG-3′) and the reverse primer *806R* (5′-GGACTACHVGGGTWTCTAAT-3′), each containing a unique barcode sequence. Paired-end sequencing data underwent quality assessment via fastp software (https://github.com/OpenGene/fastp, version 0.19.6), followed by sequence assembly using Flash software (http://www.cbcb.umd.edu/software/flash, version 1.2.11). Further analytical processes were carried out using the NovoMagic bioinformatics platform (https://magic-plus.novogene.com/#/).

### Metabolomics

Sample preparation, liquid chromatography–tandem mass spectrometry (LC-MS/MS) analysis, compound characterization, and downstream processing were conducted by Novogene (Beijing, China). Solid specimens were homogenized before collecting the supernatant into injection vials equipped with internal cannulae for instrumental analysis. Quality assurance samples were prepared by pooling equivalent volumes of metabolic extracts from all specimens. Metabolic profiling was executed using a Thermo Fisher Scientific UHPLC-Q Exactive HF-X platform coupled with mass spectrometry. Post-acquisition, raw LC-MS data underwent processing through Waters Corporation’s Progenesis QI software (Milford, USA) for baseline correction, peak detection, retention time alignment, and feature integration. A comprehensive data matrix incorporating chromatographic retention parameters, molecular mass-to-charge ratios, and spectral intensities was subsequently constructed. Metabolite annotation involved cross-referencing against established biochemical repositories including HMDB (http://www.hmdb.ca/) and METLIN (https://metlin.scripps.edu/). Statistical evaluation and data visualization were accomplished through the NovoMagic cloud platform (https://magic-plus.novogene.com/#/).

### Enzyme-Linked Immunosorbent Assay

The antibody was mixed with coating solution to achieve a protein content ranging from 1 to 10 μg/ml. Each well received 0.1 ml of the prepared test solution, followed by a 1-h incubation period at 37 °C. Post-incubation, all wells underwent thorough rinsing procedures. Subsequently, 0.1 ml of enzyme-conjugated antibody solution (previously optimized through serial dilution) was introduced into each well, with subsequent incubation maintained at 37 °C for 30 to 60 min before another washing cycle. For colorimetric detection, 0.1 ml of freshly made 3,3′,5,5′-tetramethylbenzidine (TMB) chromogenic solution was dispensed into each well, allowing chromogenic development at 37 °C for 10 to 30 min. The enzymatic reaction was terminated through addition of 0.05 ml of 2 M sulfuric acid per well. Ultimately, absorbance measurements at 450-nm wavelength were recorded for quantitative analysis across all test wells.

### Reverse transcription quantitative polymerase chain reaction

Total RNA was isolated from cellular samples and murine brain tissues employing an RNA extraction kit in accordance with guidelines provided by the manufacturer. For cDNA synthesis, ribonuclease (RNase)-free PCR tubes were utilized to conduct reverse transcription reactions containing RNA templates, reverse transcriptase enzyme, and specific primers. The qPCR preparation involved sequential addition of reaction mix, primer pairs, diethyl pyrocarbonate (DEPC)-treated water, synthesized cDNA, and supplementary reagents, with careful attention to thorough homogenization without residual droplets on tube surfaces. The reaction mixture was briefly centrifuged and mixed to eliminate any bubbles. The prepared reaction solution was then transferred to the real-time qPCR instrument, where the corresponding temperature cycling and acquisition modes were set. Fluorescence intensity measurements were recorded throughout the amplification cycles, with subsequent data interpretation employing the −ΔΔCt analytical approach for relative quantification. Primer nucleotide sequences used in these amplifications are detailed in Table S2.

### Cell lines and treatments

The BV-2 and SN4741 cell lines were maintained in high-glucose DMEM (Dulbecco’s modified Eagle’s medium) containing 10% fetal bovine serum (FBS), along with penicillin (100 U/ml) and streptomycin (100 μg/ml). A humidified environment of 37 °C and 5% CO₂ was used for cell incubation, with complete medium renewal occurring daily. Subculturing procedures were conducted every 2 d. Upon achieving 60% to 70% confluency, cells were transitioned to serum-deprived medium and exposed to varying KYNA concentrations. Inflammatory and toxic responses were triggered through administration of either 1 μg/ml LPS or 1 mM 1-methyl-4-phenylpyridinium (MPP^+^).

For siRNA transfection, siRNA was centrifuged and diluted to a 20 μM stock solution using RNase-free H₂O or sterilized ddH₂O. The siRNA sequence used is as follows: GPR35 siRNA-1 sense 5′-CAUUGUGCCUGACUUUAUA(dTdT)-3′, antisense 5′-TTCGATGCCAGTCGTGC(dTdT)-3′; GPR35 siRNA-2 sense 5′-AGGAGCACCCGGCACAAUUUC(dTdT)-3′, antisense 5′-GAAAUUGUGCCGGGUGCUCCU(dTdT)-3′; GPR35 siRNA-3 sense 5′-CCACAAAAGCCAGGACUCU(dTdT)-3′, antisense 5′-AGAGUCCUGGCUUUUGUGG(dTdT)-3′. Twenty-four hours before transfection, cells were plated to reach 30% to 50% confluency at the time of treatment. For the transfection process, 1.25 μl of siRNA (20 μM stock solution) was diluted in 25 μl of minimal essential medium (Opti-MEM, or DMEM without antibiotics and serum) and combined with 500 ng of plasmid DNA. The mixture was gently pipetted 3 to 5 times to ensure thorough mixing, after which 1.0 μl of EpFed Transfection Reagent (EpFed Biotechnology, Beijing, China) was added. The solution was again gently mixed by pipetting 3 to 5 times, avoiding vortexing or centrifugation. This protocol achieved a final siRNA concentration of 50 nM in cultured cells, followed by a 20-min room temperature incubation period.

For transfection experiments conducted in 24-well plates, each well received 25 μl of a prepared complex comprising transfection reagent combined with siRNA or plasmid DNA, which was distributed uniformly across the culture surface. The plate was then incubated at 37 °C with 5% CO₂ for 48 to 72 h. Gene silencing efficiency was assessed 48 to 72 h post-transfection.

### Western blottings

Protein samples were isolated from animal-derived tissues and cellular cultures (BV-2 and SN4741). Tissue specimens and cell pellets were chemically disrupted using NP-40 Lysis Buffer, after which the homogenates were centrifuged at 12,000*g* for 10 min under refrigerated conditions (4 °C). Protein levels were quantified with the Pierce BCA Protein Assay Kit (Thermo Fisher Scientific) following standardized protocols. Subsequently, protein aliquots were mixed with β-mercaptoethanol–containing solution, dissolved in 5× sodium dodecyl sulfate (SDS) loading buffer, and normalized to 20 μg per well for electrophoretic analysis. Thermal denaturation was performed by heating samples at boiling temperature (100 °C) for 5 to 7 min prior to electrophoretic separation. Electrophoresis was conducted using SDS-polyacrylamide gels, followed by protein transfer to polyvinylidene fluoride (PVDF) membranes using standard immunoblotting techniques. Membranes were initially saturated for 120 min with tris-buffered saline with Tween 20 containing 5% nonfat dairy powder. Primary antibody incubation (targeting iNOS and COX-2; refer to the Supplementary Materials) proceeded overnight at 4 °C, succeeded by exposure to horseradish peroxidase-linked secondary antibodies. Detection was achieved through enhanced chemiluminescence substrate reaction, with chemiluminescent signals captured using a Tanon G2500 imaging system (Tanon, Shanghai) following appropriate exposure durations.

### Detection of oxidative stress markers

Oxidative stress markers were assessed according to the protocols provided by the respective assay kits: the mitochondrial membrane potential assay kit with JC-1, the Total Antioxidant Capacity Assay Kit (FRAP method, S0116, Beyotime), the Reactive Oxygen Species Assay Kit (S0033S, Beyotime), the Total Superoxide Dismutase Assay Kit (WST-8 method, S0101S, Beyotime), the Catalase Assay Kit (S0051, Beyotime), the reduced glutathione and oxidized glutathione disulfide (GSSG) Assay Kit (S0053, Beyotime), and the Lipid Peroxidation Assay Kit (S0131S, Beyotime). Measurements were performed using an iMark Microplate Absorbance Reader (Bio-Rad Laboratories, Hercules, CA, USA). The sample quantity was determined according to the precise instructions provided with each kit.

### Cell viability assay

The CCK-8 assay was employed to evaluate cell viability. BV-2 and SN4741 cells were plated into 96-well microplates, allocating 5 to 10 wells per experimental group. Following a 24-h incubation period, cellular populations were categorized into control and treatment cohorts, where the latter received different drug dosage levels. Medium replacement was performed using fresh culture solution containing 10% CCK-8 reagent. Microplates underwent 1 to 4 h of incubation. Optical density readings were recorded hourly at 450 nm, using 630 nm as the reference wavelength. Viability percentages were determined through the following formula: (Experimental group OD value ÷ Control group OD value) × 100%.

### LDH activity

Cells were plated in a 96-well plate at a concentration of 5 × 10^5^ cells/ml, with each well receiving 100 μl of cellular suspension. Experimental compounds were subsequently introduced into designated wells, and a control group was included for comparison. The plates underwent incubation in a culture chamber for 1 to 5 h. Post-incubation, the medium was carefully removed before adding 100 μl of LDH assay working solution per well. The mixture was incubated under light-protected conditions for 5 to 30 min to facilitate the reaction. Following this, 50 μl of termination reagent was dispensed into each well to terminate the enzymatic activity. Optical density values were subsequently recorded at wavelengths of 490 or 565 nm using a plate spectrophotometer for subsequent cytotoxicity assessment.

### Transmission electron microscopy

The culture medium was removed, and cellular fixation was performed using 2.5% glutaraldehyde solution maintained at 4 °C for 15 min. Following detachment, the cells were pelleted via centrifugation while preserving the fixation solution. The resulting cellular aggregates were subsequently maintained under refrigeration at 4 °C. A series of four 15-min washing cycles with 0.1 M PBS was implemented. Subsequent secondary fixation involved treatment with 1% osmium tetroxide prepared in 0.1 M PBS (pH 7.2) under ambient conditions (20 °C) for a 4-h duration. Triple washing procedures employing PBS were conducted, each cycle maintained for 15 min. Sequential dehydration processes were executed using ethanol and acetone solutions, followed by cellular permeabilization achieved through combined acetone and epoxy resin treatment.

The treated specimens were positioned in encapsulation containers or embedding chambers, with subsequent introduction of epoxy-based embedding medium. Polymerization processing was implemented through thermal curing at 60 °C maintained for 48 h. Post-curing preparation involved microtome sectioning to achieve 70-nm thin slices, which subsequently underwent dual-staining protocol: Initial treatment with uranyl acetate (2% aqueous saturation concentration) succeeded by lead citrate application, both conducted under ambient conditions for 15-min durations. Section dehydration was accomplished through natural air-drying over 24-h period at standard laboratory temperature. Ultrastructural examination was ultimately performed utilizing TEM equipment.

### Proteomics by LC-MS/MS

Following cell scraping, a lysate solution (8 M urea with 1% SDS supplemented with protease inhibitors) was introduced to resuspend the cellular material. The combined sample underwent mechanical disruption through 3 iterative 40-s treatments in a high-throughput tissue homogenizer. Quantitative analysis of protein content was conducted via bicinchoninic acid assay (BCA) method. Proteins then underwent sequential reduction, alkylation, and enzymatic digestion processes. Final peptide concentrations were measured according to manufacturer protocols specified in the Peptide Quantification Kit (Thermo Fisher Scientific, catalog no. 23275), employing established spectrophotometric techniques.

Following dissolution of peptide samples in appropriate mass spectrometry buffer, analytical separation and detection were performed using an EASY-nLC 1200 nanoflow liquid chromatography system paired with a Q Exactive HF-X hybrid quadrupole-orbitrap mass spectrometer in MS/MS mode. All raw spectral data were subsequently transferred to the Majorbio cloud-based analysis platform (accessible at cloud.majorbio.com) for comprehensive processing. Intergroup comparative analysis involving both statistical significance (expressed as *P* values) and quantitative fold-change differences was executed through implementation of Student’s *t* test methodology.

### RNA sequencing

Total RNA was isolated from cellular specimens, with quantification of nucleic acid concentration and assessment of purity conducted via NanoDrop 2000 spectrophotometric analysis (Thermo Fisher Scientific, Waltham, MA, USA). Electrophoretic separation on agarose matrices was employed to evaluate RNA integrity, complemented by RNA quality number (RQN) determination using the Agilent 5300 analytical platform. Oligo(dT)-functionalized magnetic beads facilitated selective mRNA enrichment through complementary A–T base pairing with polyadenylated termini, enabling effective separation from ribosomal and noncoding RNA species. Enzymatic fragmentation protocols were implemented to generate randomly distributed RNA segments averaging 300 base pairs. First-strand cDNA synthesis was initiated using reverse transcriptase, followed by second-strand synthesis to produce double-stranded DNA fragments. Terminal repair reactions utilizing specialized enzyme mixtures converted cohesive ends to blunt termini, succeeded by 3′-terminal adenosine addition to facilitate adapter ligation. Post-ligation purification processes eliminated unbound adapters, with subsequent size selection optimizing fragment distributions. Amplification through thermocyclic PCRs yielded sequencing-ready libraries, which underwent final quality control assessments prior to high-throughput sequencing on the NovaSeq X Plus instrumentation (Illumina, San Diego, CA, USA).

### Statistical analysis

To determine the significance of differences between experimental groups, statistical analyses were conducted. A one-way analysis of variance (ANOVA) was utilized for comparing means ± SEM obtained from 3 separate experiments. A significance threshold of ^*^P < 0.05 was applied to identify statistical differences. All statistical evaluations were carried out with GraphPad Prism 10 (GraphPad Software, San Diego, CA, USA).

## Data Availability

Data are available upon reasonable request. All data are available in the main text or Supplementary Materials. Correspondence and requests for materials should be addressed to S.M. (ma.shaohua@sz.tsinghua.edu.cn) on reasonable request.
