# Supplementary material for: Gpr35 Expression Mitigates Neuroinflammation and Enriches Gut Lactobacillus to Relieve Parkinson’s Disease
Source: Research (Wash D C). 2025 Aug 25;8:0846. doi: 10.34133/research.0846 (PMC12376290; doi:10.34133/research.0846)
Supplement: Supplementary 1 — Tables S1 and S2 Figs. S1 to S8 [file research.0846.f1.docx]

**Supplemental material**

**Supplementary Tables**

**Supplementary Table 1.** Chemicals and reagents

| **REAGENT or RESOURCE** | **SOURCE** | **IDENTIFIER** |
| --- | --- | --- |
| Antibodies | | |
| Anti-Tryptophan Hydroxylase Antibody | Sigma-Aldrich | #T0678 |
| Anti-Iba1 antibody [EPR16588] | abcam | #ab178846 |
| Anti-iNOS antibody [EPR16635] | abcam | #ab178945 |
| Anti-Liver Arginase antibody | abcam | #ab96183 |
| Anti-NeuN antibody [EPR12763] - Neuronal Marker | abcam | #ab177487 |
| Anti-Caspase-3 antibody [E87] | abcam | #ab32351 |
| Anti-Claudin 5 antibody [EPR7583] | abcam | #ab131259 |
| Anti-MAP2 antibody [EPR19691] | abcam | #ab183830 |
| Chemicals | | |
| Lipopolysaccharides from Escherichia coli O111:B4 Ready Made solution, 1 mg/mL | Sigma-Aldrich | #L5293 |
| MPP+ iodide | Sigma-Aldrich | #D048 |
| Kynurenic acid 98% | Sigma-Aldrich | #H58609 |
| MPTP Hydrochloride | Sigma-Aldrich | #M0896 |
| Critical commercial assays | | |
| Eastep® Super Total RNA Extraction Kit | Promega | #LS1040 |
| Cell Counting Kit 8 (WST-8 / CCK8) | abcam | #ab228554 |
| Pierce BCA Protein Assay Kit | Thermo Scientific™ | #23227 |
| Total Antioxidant Capacity Assay Kit with FRAP method | Beyotime Biotech Inc | #S0116 |
| *Continued* |  |  |
| **REAGENT or RESOURCE** | **SOURCE** | **IDENTIFIER** |
| Critical commercial assays | | |
| Total Superoxide Dismutase Assay Kit with WST-8 | Beyotime Biotech Inc | #S0101 |
| Catalase Assay Kit | Beyotime Biotech Inc | #S0051 |
| GSH and GSSG Assay Kit | Beyotime Biotech Inc | #S0053 |
| Lipid Peroxidation MDA Assay Kit | Beyotime Biotech Inc | #S0131 |
| Mouse IL-1β ELISA Kit | Wuhan Saipei Biotechnology Co., Ltd | #SP12667 |
| Mouse Tumor necrosis factor α,ELISA KIT | Wuhan Saipei Biotechnology Co., Ltd | #SP13726 |
| Mouse Interleukin 6,IL-6 ELISA KIT | GIELD BIOTECHNOLOGY | #J24111 |
| Experimental models: Cell lines | | |
| Mouse Microglia Cells ,bv-2 | GAINING BIOLOGICAL | #CM-M059 |
| SN4741 | EDITGENE | #EDC2024014-W014 |
| Experimental models: Organisms/strains | | |
| C57BL/6J | Changsheng Biotechnology | N/A |
| C57BL/6Smoc-Gpr35em1Smoc | Shanghai Model Organisms | #NM-KO-2106644 |
| *Lactobacillus* | Ningbo Mingzhou Biotechnology Co., Ltd. | N/A |
| (Gpr35 siRNA) | GenePharma | N/A |
| (Gpr35 siRNA) | GenePharma | N/A |
| Primers for mouse brain samples quantitative  real-time PCR, see Table S1 | Sangon Biotech | N/A |
| Software and algorithms | | |
| Image J | NIH | https://imagej.net/ij/ |
| Prism 10 | GraphPad software | https://www.graphpad-prism.cn/ |
| *Continued* |  |  |
| **REAGENT or RESOURCE** | **SOURCE** | **IDENTIFIER** |
| Software and algorithms | | |
| Adobe Illustrator CS6 | Adobe System | N/A |
| ANY-maze | Stoelting | https://satijalab.org/seurat/ |
| R studio (3.5) | R software | https://www.rstudio.com/ |

**Supplementary Table 2.** Primer Sequences

| **Primer** | **Forward** | **Reverse** |
| --- | --- | --- |
| *Gpr35* | AGTACAACCTGTAACAGCACC | GCAGTGTGTGTGGCCCTTTG |
| *IL-6* | CCAGAAACCGCTATGAAGTTCC | GTTGGGAGTGGTATCCTCTGTGA |
| *IL-1β* | GTTCCCATTAGACAACTGCACTACAG | GTCGTTGCTTGGTTCTCCTTGTA |
| *TNF-α* | CCCCAAAGGGATGAGAAGTTC | CCTCCACTTGGTGGTTTGCT |
| *GCLM* | ATGACCCGAAAGAACTGCTCTCTG | GCTCTTCACGATGACCGAGTACC |
| *CAT* | GCGTCCAGTGCGCTGTAGA | TCAGGGTGGACGTCAGTGAA |
| *CCL1* | GGATGTTGACAGCAAGAGCA | TAGTTGAGGCGCAGCTTTCT |
| *Arg-1* | GAATCCCACCTAGGAGACAAAG | GTCTATACTCCCTGCAGTTTCC |
| *COX-2* | TGAGTGGTAGCCAGCAAAGC | CTGCAGTCCAGGTTCAATGG |
| *iNOS* | GTTCTCAGCCCAACAATACAAA | GTGGACGGGTCGATGTCAC |
| *CD206* | TGAGCTGTTTTGGTTGGGAC | CCCATCTGCAGTAACTGGTG |
| *FIZZ-1* | CCAATCCAGCTAACTATCCCTCC | ACCCAGTAGCAGTCATCCCA |
| *YM-1* | TGAATGAAGGAGCCACTGAG | TTGTTGTCCTTGAGCCACTG |
| *GCLC* | GATGTGGACACCCGATGCAG | CAGGATGGTTTGCAATGAACTCTC |
| *IL-10* | GGCAGAGAACCATGGCCCAGAA | AATCGATGACAGCGCCTCAGCC |
| *CCL5* | CTGCTGCTTTGCCTACCTCT | CGAGTGACAAACACGACTGC |
| *CCL3* | AAGGATACAAGCAGCAGCGAGTA | TGCAGAGTGTCATGGTACAGAGAA |

**Supplementary Figures**

**Supplementary Figure 1**

**
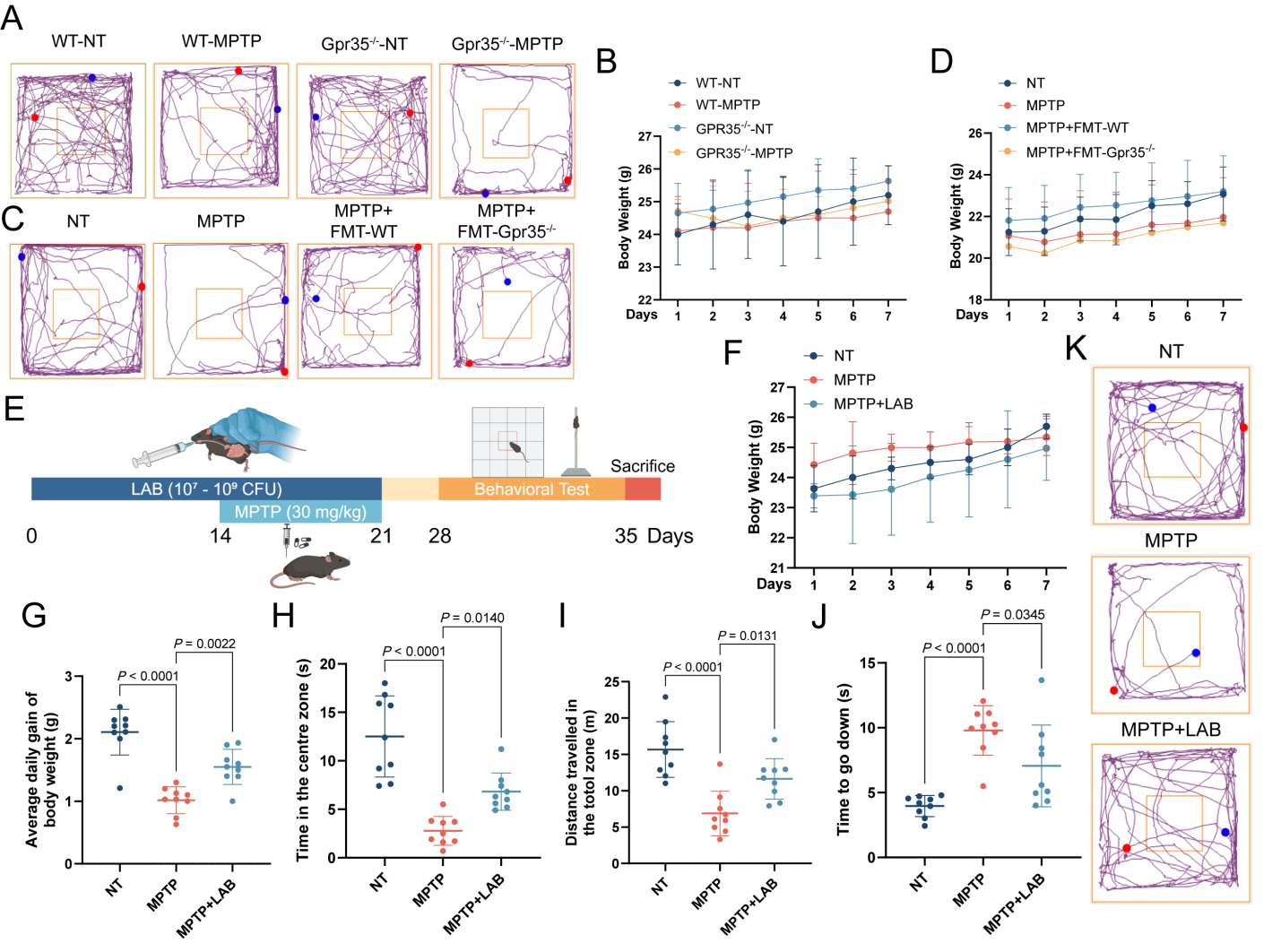
**

**Supplementary Figure 1.** Behavioral characterization of PD mice. (A, C and K)Representative movement trajectories of mice in the open-field test. (B, F and D) Body weight changes of WT and Gpr35^-/-^ mice monitored from day 0 to day 7. (E) Flow chart of *Lactobacillus* (*LAB*) determination experiments (n=9 biologically independent mice). (G) The average daily weight gain during the observation period was quantified (n=9 biologically independent mice). (H) The time mice spent crossing the central zone in the open-field test was recorded (n=9 biologically independent mice). (I) The total distance traveled in the open-field test was also quantified (n=9 biologically independent mice). (J) Time required for mice to climb down from the top during the pole test (n=9 biologically independent mice). Data are presented as the mean ± SEM. Data represent positive spots from three independent experiments.

**Supplementary Figure 2**

**
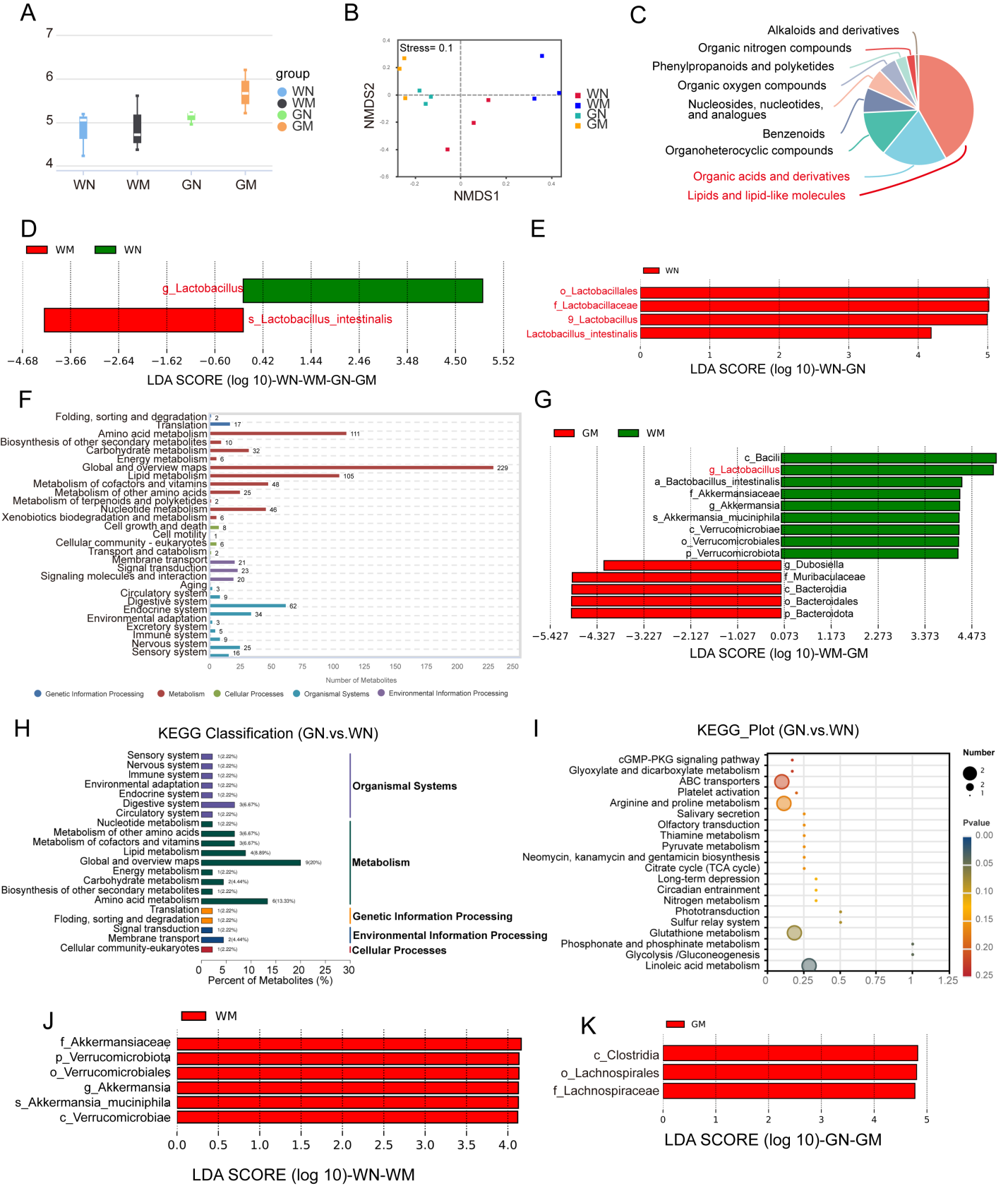
**

**Supplementary Figure 2.** Structural and compositional analysis of intestinal flora and metabolites. (A) Shannon - Wiener index illustrating the α-diversity of microbial communities across experimental groups (n=3 biologically independent mice). (B) β-diversity analysis of gut microbiome from cecal contents among four experimental groups, determined by NMDS based on Bray-Curtis distances (n=3 biologically independent mice). (C) Pie chart showing the proportion of all classified metabolites (based on the ClassyFire database). (D) LDA scores of differentially enriched bacterial genera obtained from LDA Effect Size (LEfSe) analysis of all four groups. (E) LDA scores of differentially enriched bacterial genera obtained from LEfSe analysis between the WN and GN mice. (F) Functional annotation of all detected metabolites using KEGG pathway analysis. (G) LDA scores of differentially enriched bacterial genera identified through LEfSe analysis comparing WM and GM groups. (H-I) KEGG classification (F) and enrichment analysis (G) of differential metabolites between GN vs. WN. (J) LDA scores of differentially enriched bacterial genera identified through LEfSe analysis comparing WN and WM groups. (K) LDA scores of differentially enriched bacterial genera identified through LEfSe analysis comparing GN and GM groups. Data are presented as the mean ± SEM.

**Supplementary Figure 3**

**
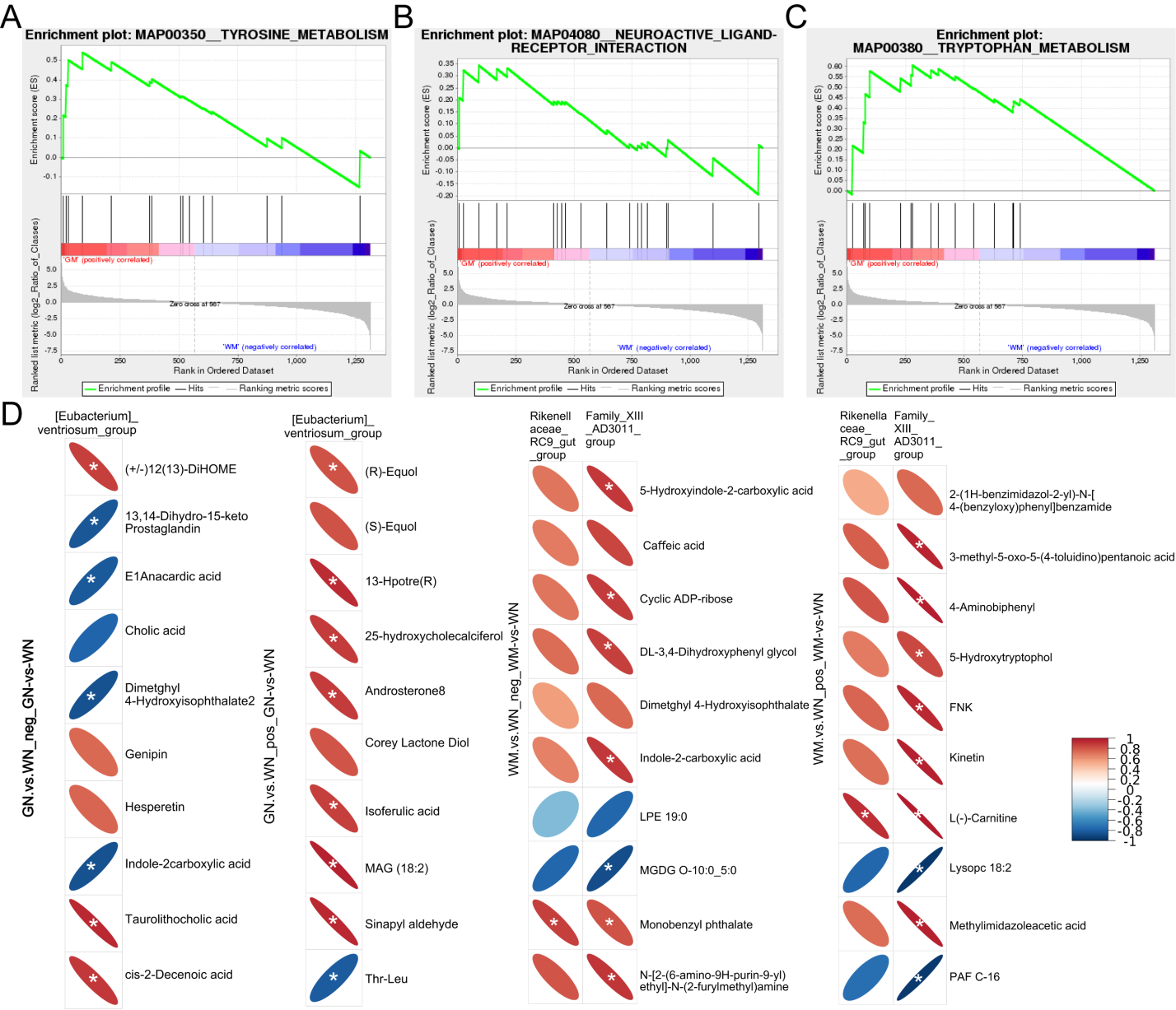
**

**Supplementary Figure 3.** Integrated Analysis of Gut Microbiome Sequencing and Metabolomics. (A-C) GSEA enrichment plot of differential metabolites. (D) The heatmap of correlation analysis illustrates the associations between distinct differentially abundant bacterial genera and metabolites. The legend indicates the correlation coefficient values, where deeper red hues indicate stronger positive correlations, and deeper blue hues signify stronger negative correlations. The flatness of the ellipses reflects the magnitude of the correlation absolute value, with flatter ellipses indicating stronger correlations. Data are presented as mean ± S.D. Data represent positive spots from three independent experiments, with n=3 per group pooled. *^*^P* <0.05.

**Supplementary Figure 4**

**
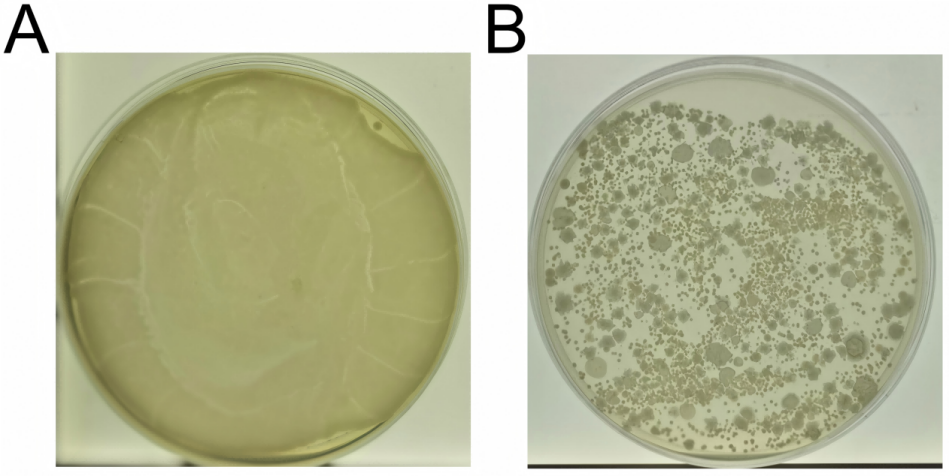
**

**Supplementary Figure 4.** Culturing of fecal microbiota from mice treated with and without antibiotic cocktail. (A) Removal of bacterial groups: No obvious bacterial colonies were seen on the fecal coating plate; only large patches of plaque were successfully removed. (B) Not cleared bacterial groups: obvious bacterial colonies can be seen on the fecal coating plate.

**Supplementary Figure 5**

**
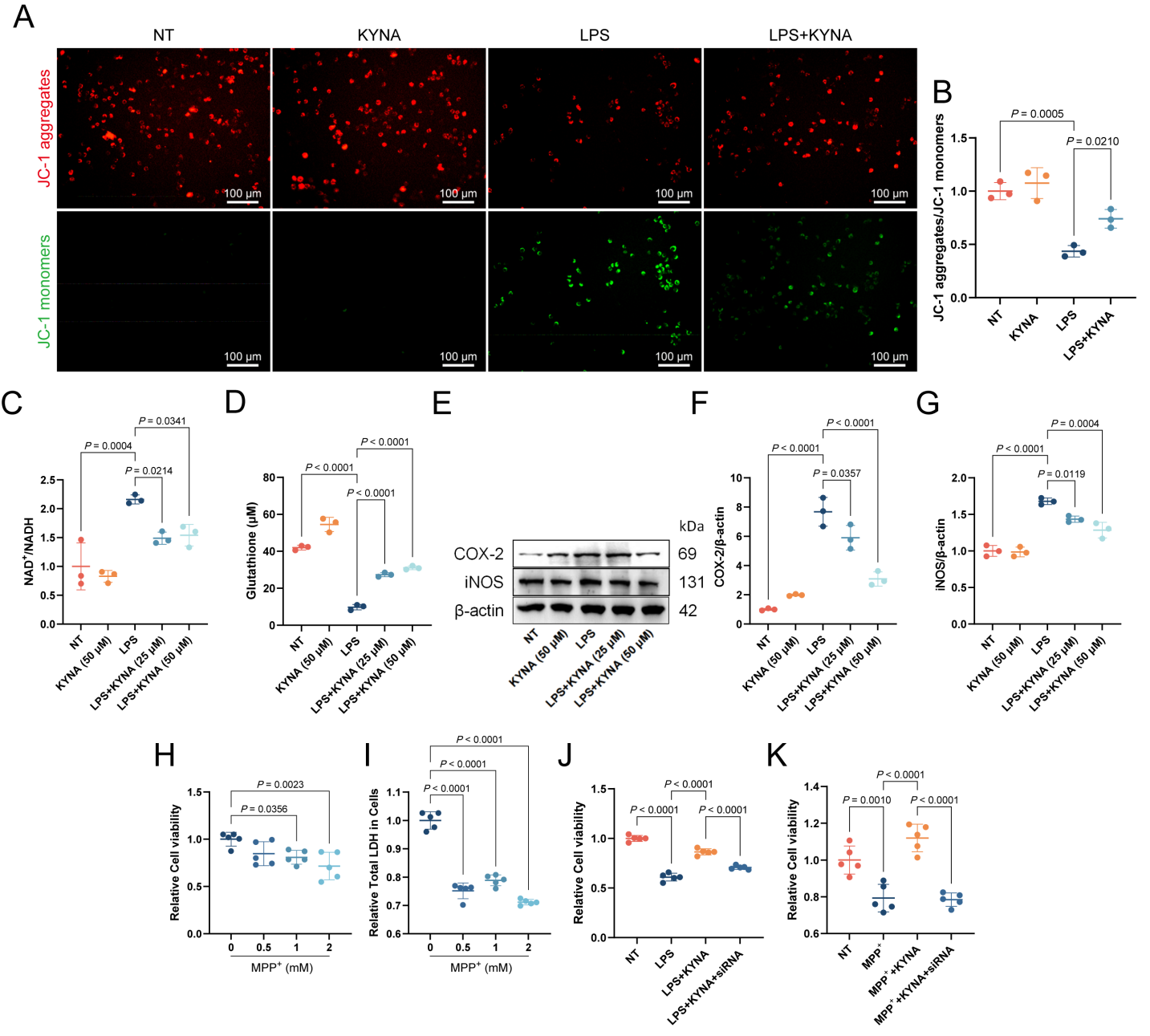
**

**Supplementary Figure 5.** Effect of the Gpr35 agonist KYNA on mouse microglial and dopaminergic neuronal cells. (A and B) Immunofluorescence staining of JC-1 aggregates (red) and monomers (green) in the BV-2 cell line after LPS induction with or without KYNA treatment. The relative fluorescence intensity (B) was quantified. (C and D) The ratio of NAD^+^/NADH (C) and the concentration of glutathione (D) in BV-2 cells. (E-G) Protein levels of iNOS (E, G) and COX-2 (E, F) in BV-2 cells. (H) Cell viability of SN4741 cells exposed to varying MPP^+^ concentrations, assessed using the CCK-8 assay. (B) Cell viability of SN4741 cells exposed to varying MPP^+^ concentrations, evaluated by LDH assay. (C) Cell viability of neuronal cells following KYNA and siRNAtreatment, analyzed by CCK-8 assay. (D) Cell viability of SN4741 cells after BV-2 cells co-culture, determined by CCK-8 assay. Data are presented as the mean ± SEM. Data represent positive spots from three independent experiments, with n=5 per group pooled.

**Supplementary Figure 6**

**
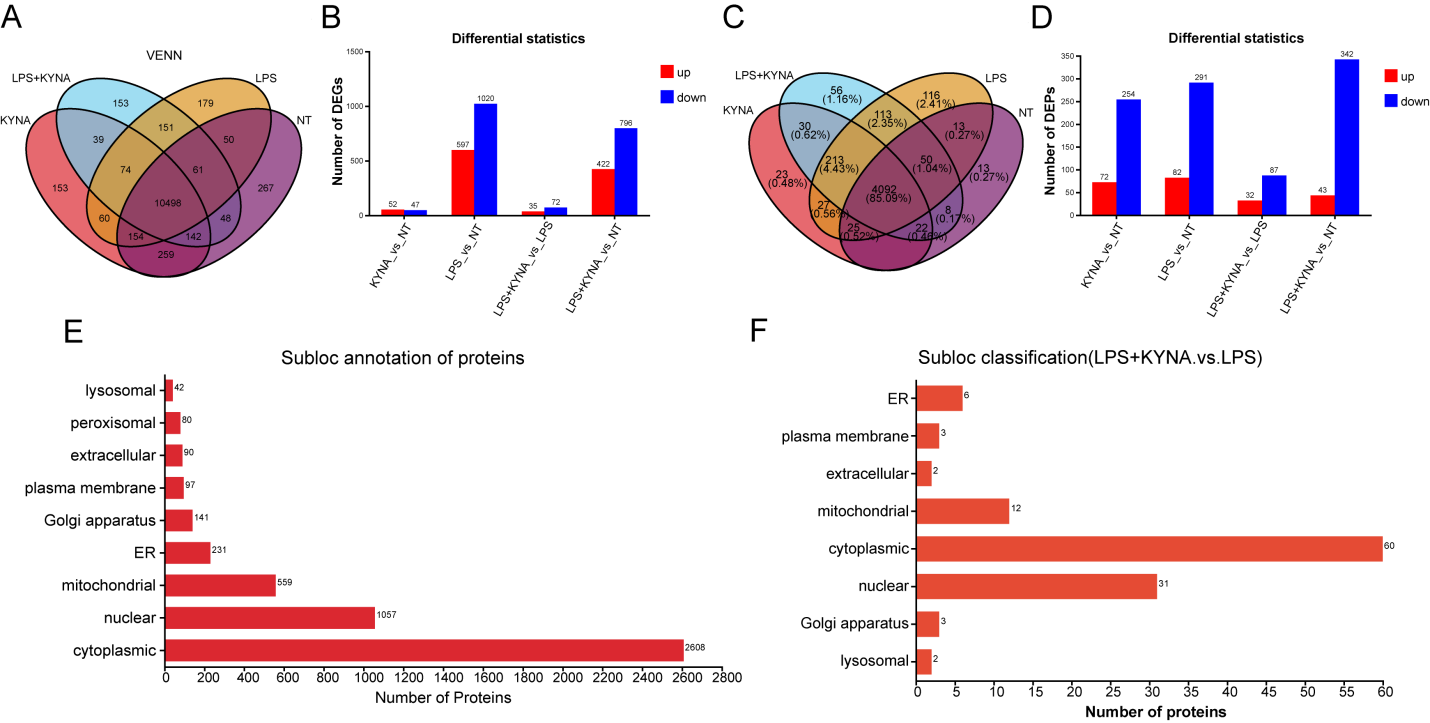
**

**Supplementary Figure 6.** Proteomic profiling and functional annotation analysis based on mass spectrometry identification. (A) Venn diagram display DEGs across four groups. (B) The number of DEGs. The vertical axis indicates the number of upregulated and downregulated genes between two groups. (C) Venn diagram display DEPs across four groups. (D) The number of DEPs between groups. (E) Subcellular localization analysis of identified proteins. (F) Subcellular localization of DEPs between the LPS group and the LPS+KYNA group. Data are presented as the mean ± SEM. Data represent positive spots from three independent experiments, with n=3 per group pooled.

**Supplementary Figure 7**

**
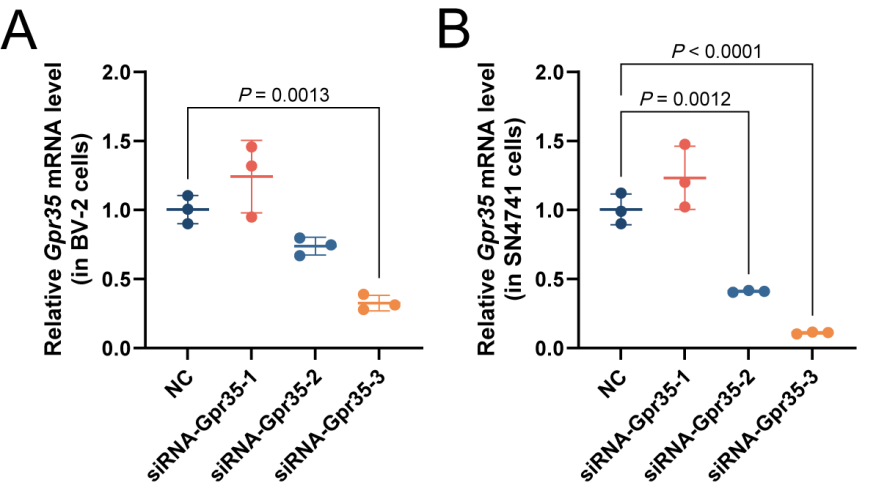
**

**Supplementary Figure 7.** Relative mRNA expression of *Gpr35* normalized to β-actin and calculated relative to NC siRNA group. (A) Relative mRNA expression of *Gpr35* in BV-2 cells. (B) Relative mRNA expression of *Gpr35* in SN4741 cells. Data are presented as mean ± SEM.

**Supplementary Figure 8**

**
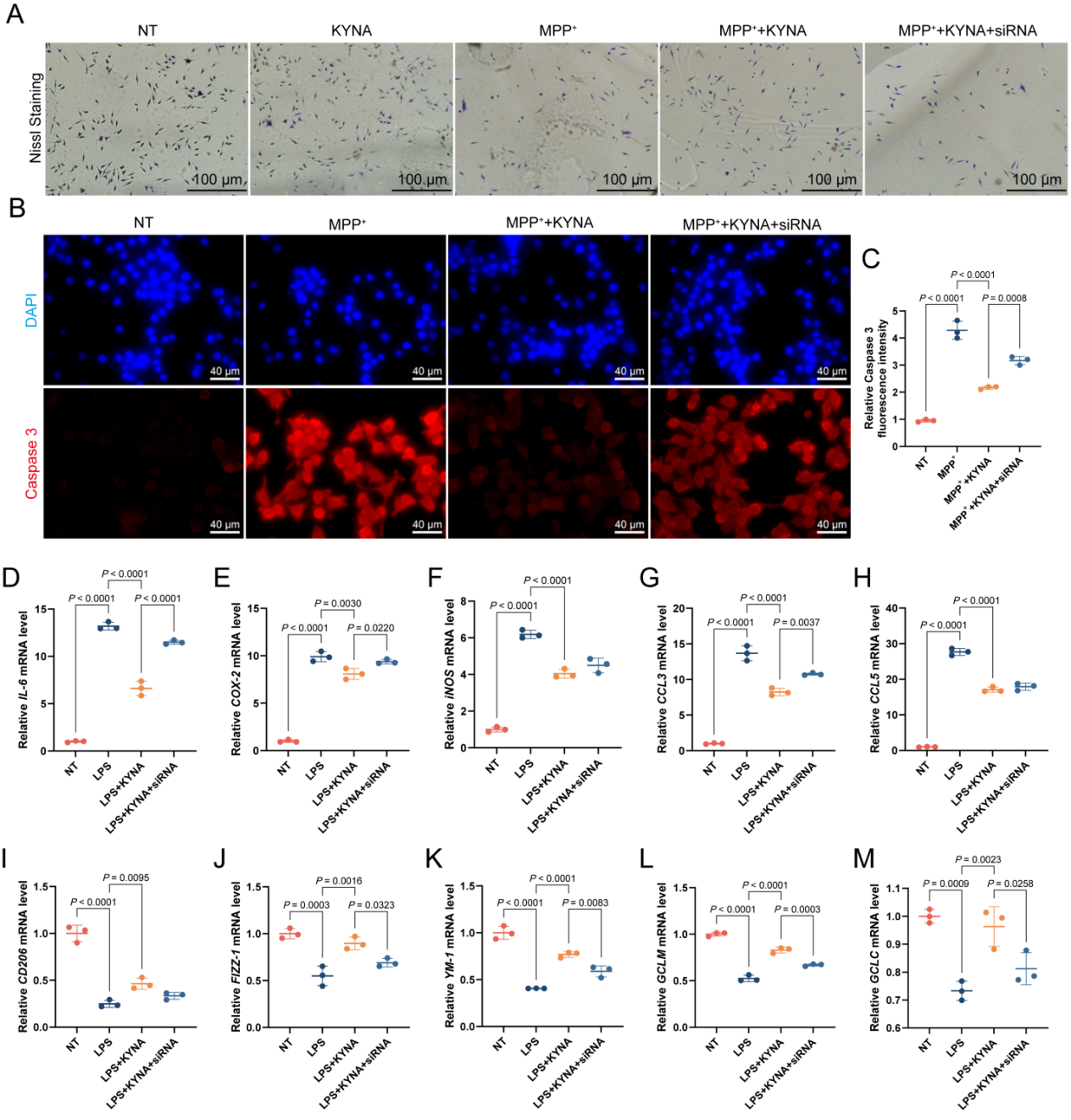
**

**Supplementary Figure 8.** Impact of GPR35 siRNA Knockdown on Microglial and Neuronal Cells. (A) Nissl staining of SN4741 cells following MPP^+^ stimulation and treatment under different conditions. (B-C) Representative immunofluorescence images of Caspase 3 in the dopaminergic neuronal cell line SN4741, with quantification of the mean fluorescence intensity shown in (C). (D-M) RNA expression of inflammatory cytokines (*IL-6*, *COX-2*, *iNOS*, *CCL3*, *CCL5*, *CD206*, *FIZZ-1*, *YM-1*, *GCLM*, and *GCLC*) in BV-2 microglial cells. Data are pooled from at least two independent experiments and presented as mean ± SEM.
